# Effects of large herbivore grazing on relics of the presumed mammoth steppe in the extreme climate of NE-Siberia

**DOI:** 10.1038/s41598-021-92079-1

**Published:** 2021-06-21

**Authors:** Jennifer Reinecke, Kseniia Ashastina, Frank Kienast, Elena Troeva, Karsten Wesche

**Affiliations:** 1 Senckenberg Museum of Natural History, Görlitz, Germany; 2 International Institute Zittau, Technische Universität Dresden, Markt 23, 02763 Zittau, Germany; 3 Senckenberg Research Institute and Natural History Museum, Research Station of Quaternary Paleontology, Weimar, Germany; 4 Institute of Ecology and Evolution, Friedrich Schiller University Jena, Philosophenweg 16, 07743 Jena, Germany; 5 Institute for Biological Problems of Cryolithozone, Siberian Branch of RAS, Yakutsk, Russia; 6 German Centre for Integrative Biodiversity Research (iDiv) Halle-Jena-Leipzig, Leipzig, Germany

**Keywords:** Ecology, Plant sciences, Systems biology, Ecology, Environmental sciences

## Abstract

The Siberian mammoth steppe ecosystem changed dramatically with the disappearance of large grazers in the Holocene. The concept of Pleistocene rewilding is based on the idea that large herbivore grazing significantly alters plant communities and can be employed to recreate lost ecosystems. On the other hand, modern rangeland ecology emphasizes the often overriding importance of harsh climates. We visited two rewilding projects and three rangeland regions, sampling a total of 210 vegetation relevés in steppe and surrounding vegetation (grasslands, shrublands and forests) along an extensive climatic gradient across Yakutia, Russia. We analyzed species composition, plant traits, diversity indices and vegetation productivity, using partial canonical correspondence and redundancy analysis. Macroclimate was most important for vegetation composition, and microclimate for the occurrence of extrazonal steppes. Macroclimate and soil conditions mainly determined productivity of vegetation. Bison grazing was responsible for small-scale changes in vegetation through trampling, wallowing and debarking, thus creating more open and disturbed plant communities, soil compaction and xerophytization. However, the magnitude of effects depended on density and type of grazers as well as on interactions with climate and site conditions. Effects of bison grazing were strongest in the continental climate of Central Yakutia, and steppes were generally less affected than meadows. We conclude that contemporary grazing overall has rather limited effects on vegetation in northeastern Siberia. Current rewilding practices are still far from recreating a mammoth steppe, although large herbivores like bison can create more open and drier vegetation and increase nutrient availability in particular in the more continental Central Yakutian Plain.

## Introduction

In view of the current massive decline of biodiversity and ecosystem functioning in the Anthropocene (sensu Doughty^1,2^), the idea of rewilding sparks ongoing interest among paleoecologists, conservation scientists and practitioners. “Pleistocene rewilding” aims at reconstructing pre-historic megafaunas, and is based on expected large top-down effects on the entire ecosystem^2–7^. There is indeed evidence that large herbivores have shaped vegetation structure, ecosystem processes, landscape heterogeneity and even entire biomes in their time^8–10^. This prompted several authors to propose alternative climax states for contemporary vegetation, if herbivores would still play a major role (e.g. “Steppenheidentheorie”^11^; “wood-pasture theory”^4^, „Alternative Biome States"^12^). Zimov et al.^5,13–15^ have developed a herbivore-vegetation-model (“Ecosystem hypothesis”), claiming that a cold steppe, which once existed under dry-cold Pleistocene glacial stages^16–18^, would still be the natural vegetation of NE-Siberia today.

During Pleistocene cold stages, the non-glaciated northern latitudes were characterized by extensive herb-dominated grassland vegetation that supported a wide array of large herbivores in a now-extinct cold steppe biome—the mammoth steppe^17,19^ (see Appendix S1: Table S1_1 for details). According to pollen and macrofossil analysis, the Pleistocene mammoth steppe was characterized by typical steppe and tundra plants^18,20^. The most common representatives of the late Pleistocene cold-adapted mammoth fauna in northern Yakutia were woolly mammoth^21^, steppe bison and horse, the latter two primarily grazers^22–24^, and the mammoth a mixed feeder^25^. Less abundant herbivores, like saiga and woolly rhino, were also grazers and strictly restricted to steppe-like habitats^26,27^. Even typical arctic faunal elements, such as musk ox and reindeer^28^, were mixed feeders (browsing and grazing) with considerable proportions of grass in their diet^29^. Thus, according to the requirements of the mammoth fauna, cold stage vegetation must have consisted of open grassland on dry, firm ground. Grazer densities as high as 10.5 tons of herbivore biomass/1 km^2^ have been suggested^13^, but such estimates from skeleton collections are highly debated^6,21^. Productivity of vegetation should have been relatively high compared to current conditions to support high densities of large herbivores^10,19,30^, and soils should have been more fertile^19^ than today in the arctic, where nutrient deficiency is often the limiting factor^31^.

Today, wild herbivore density is relatively low, with only domestic horses, and reindeer in the north occurring in larger herds^13,14^. Higher densities of grazers are only reached in game parks, where supplementary feeding sustains these herds. The climate of Siberia is warmer and the soils are wetter and less fertile today^19^ than during Pleistocene cold stages. Vegetation biomass is mainly stored in slow-growing, inedible woody plants. The zonal vegetation is taiga or tundra, with a ground layer dominated by mosses and dwarf shrubs.

**Figure 1:**
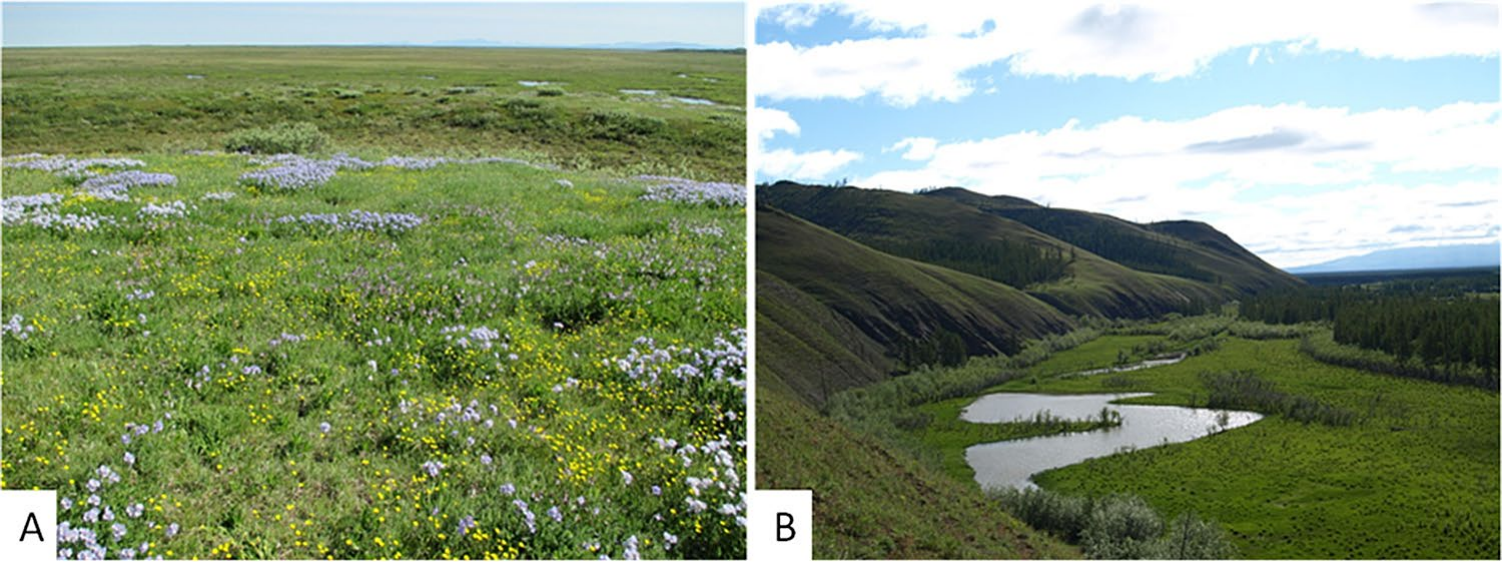
(**A**) Tundra steppe on elevations in dwarf shrub tundra close to Pokhodsk, Yakutia. (**B**) Extrazonal steppe on SW-exposed slopes close to Verkhoyansk, Yakutia. Photos by J. Reinecke.

Steppes and tundra steppes are confined to local sites with special microclimatic conditions^32^ (Fig. 1). It is still debated to which degree modern (tundra) steppes are analogues of the Pleistocene mammoth steppe^16,33–35^, or whether the lack of herbivore pressure resulted in the formation of novel plant communities^36^. We showed in previous studies that contemporary steppes resemble both modern Central Asian steppes^32^ and palaeobotanic records of the mammoth steppe, thus supporting the hypothesis of a continuation of steppes in northern Siberia throughout the late Pleistocene and Holocene^20,34^. On the other hand, the palaeobotanic record contained species, which today occur in separate grassland communities and included more disturbance indicators^34^, which indicates that the species composition of these grasslands has changed. Thus, modern steppes may not be exact analogues to the mammoth steppe and the role of herbivory as a driver of their differentiation is not yet clear.

In the given context, the Pleistocene rewilding hypothesis is that given enough time large herbivores would drive the vegetation towards openness, higher productivity and a higher prevalence of xerophytic species^15^: grazing and trampling would create disturbances in the vulnerable moss and shrub layers; droppings and urine deposition would boost nutrient turnover and fertilize the soil; productive and grazing-tolerant grasses would have a competitive advantage over dwarf shrubs and mosses. Subsequently, higher evapotranspiration of grasses would increase water uptake and cause xerophytization of vegetation. The interactions of large herbivores and grazing-adapted grass-dominated vegetation would then, over time, establish a productive grazing system in a cold and dry environment^15^. This would result in the replacement of currently dominant shrub tundra and larch taiga by more open park-like landscapes, with plant species of more xeric grasslands and dry steppes.

This hypothesis has, however, to the best of our knowledge never been assessed against empirical evidence from vegetation studies in northern Siberia. According to standard concepts of rangeland ecology, ecosystems with a long history of grazing typically have species pools adapted and resilient to different grazing intensities^37^. Grazing effects on species diversity occur, yet should be largest at moist and productive sites, while low productive sites display small effects^37^. This is confirmed by evidence from typical steppes of Mongolia and China (e.g.^38,39^). Hardly any studies exist for the extreme rangelands of northern Yakutia. There, water limitation during the summer season is less pronounced, due to moisture supply from permafrost and lacking percolation, yet low temperatures result in low-productivity and slow decomposition and nutrient cycling constraining plant growth^31,40^. Rangeland ecology would suggest small effects of grazing on nutrient-limited larch taiga and dwarf shrub tundra, as well as on dry steppe slopes, and larger effects only on nutrient-richer and moister sites, such as floodplain meadows^37^. However, large herbivores may also cause indirect effects and accelerate nutrient cycling even in the arctic, making nitrogen available for plants, and thus potentially creating positive feedback systems.

The closest analogue studies of Pleistocene vegetation in regard to grazing under harsh climates are sites with reindeer grazing in Scandinavian taiga and tundra, followed by yak grazing in Tibetan alpine meadows and meadow steppes. In lichen-rich winter pastures of Scandinavia, intensive grazing and trampling has been shown to reduce vegetation cover, eventually leading to increased bare ground and reduced productivity^41^. In contrast, moderate grazing in grass- and herb-dominated summer pastures can lead to the formation of grasslands or open parklands^42^. Changes in species composition may be strong in low productive tundra habitats^43^, as well as in productive floodplain tundra grasslands^44^. Overall, reindeer grazing effects seem to be spatially inconsistent^44^ and rather depend on environmental and grazing conditions of the respective sites^42^.

Similarly, a meta-analysis on grazing studies in Chinese grasslands showed that vegetation responses to both abiotic factors and grazing are highly variable across regions^45^. Heavy grazing usually reduces vegetation cover and biomass, while changes in species composition are often negligible in established grasslands^45^. Lighter grazing might have positive effects on vegetation cover, through facilitation of nutrient or water uptake^45^. Steppes seem to be rather tolerant to grazing and annual plants profit from soil disturbances only in moister meadows. Overall, species composition seems to be little affected by grazing, and environmental factors override grazing effects over much of Central Asia^39^.

When studying grazing responses across large spatial and environmental scales, plant functional traits can be used to generalize shifts in species composition^46–48^. A small number of traits have been found to be informative and reliable indicators for grazing responses^47^ (Appendix S1: Table S1_2). A global meta-analysis by Díaz et al.^47^ revealed that grazing favored annuals over perennials, short over tall plants, prostate growing over erect growing plants, and rosettes over stoloniferous plants and tussock grasses. Again, interactions with local climate are pronounced, with minimal to insignificant effects in dry systems with a long grazing history^47^.

Literature on rewilding is mainly dominated by essays and opinion pieces, while empirical evidence is scarce and has a strong spatial bias, with most studies focusing on North America, Europe, and oceanic islands^2^. With the Ust-Buotama Bisonary in Central Yakutia and the Pleistocene Park in the subarctic Kolyma lowland, we here make use of two rewilding projects in northeastern Siberia, which have not been studied regarding grazing effects on plant communities so far. Livestock grazing has been studied in meadow and steppe communities on shallow river terraces and alas depressions in the Central Yakutian Plain^49,50^. However, grazing studies from northeastern Yakutia (Kolyma basin; except for reindeer pastures) and across a larger climatic and environmental gradient are missing. Bison and horses, the central grazers in our study region, have Pleistocene counterparts and could thus be suitable representatives of the mammoth steppe fauna. Bison are furthermore regarded ecological keystone species, which, due to their selective grazing and wallowing, support greater diversity in structure and composition of vegetation than, e.g., domestic livestock such as cattle^51^. Focusing on current extrazonal steppe vegetation as a potential analogue to the Pleistocene mammoth steppe vegetation, we try to bridge the knowledge gap between the large herbivore hypotheses of paleoecologists, who focus mainly on paleobotanic reconstructions and paleozoological population dynamics; and current rangeland ecologists, whose theories focus mainly on herbivore–vegetation interactions in arid steppes or savannas.

We addressed the following questions.

- If extrazonal steppes are potential relics of the Pleistocene mammoth steppe, are they significantly affected by grazing today? Can grazing explain the plant community composition of steppes or even their occurrence or is climate more important?
- Given the extreme climate, does grazing significantly affect Siberian vegetation at all? How does grazing affect vegetation structure and community composition, taxonomic and functional diversity as well as productivity and chemical composition of plant biomass?
- How do different intensities and types of modern grazing animals (livestock as well as undomesticated herbivores) influence grazing effects?

## Methods

### Study area

**Figure 2:**
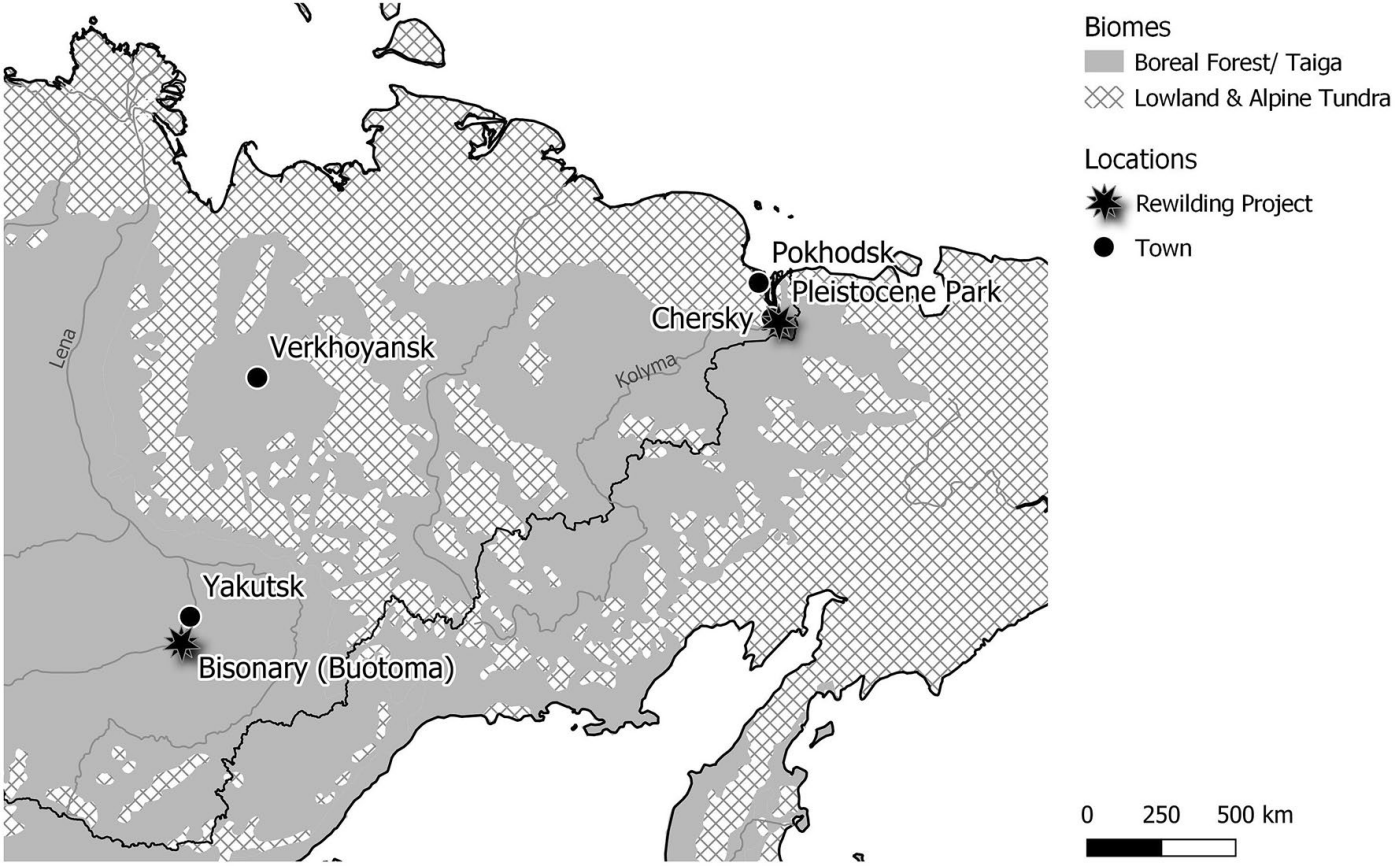
Map of the study area. Physical Map: made with Natural Earth; Biomes: adapted from https://ecoregions2017.appspot.com/.

Sakha (Yakutia) is located in northeastern Siberia and characterized by extreme climatic conditions (Table 1; Appendix S1, Figure S1_1). We collected our data in two expeditions, to the Yana highlands in June/July 2014 and to the lower Kolyma and middle Lena river basins in July/August 2015 (also see^52^). Our study region includes five locations (Table 1; Fig. 2): Pokhodsk and Chersky in the lower Kolyma river basin in northeastern Yakutia, Verkhoyansk in the Yana highlands, and Yakutsk and the Buotoma river confluence in the middle Lena river basin in Central Yakutia. We visited two fenced grazing sites, the “Pleistocene Park” in Chersky and the “Bisonary” along the Buotoma river confluence. “Pleistocene Park” was founded in 1996 with the specific aim to test effects of Pleistocene rewilding^15^ and is set in the northern taiga zone (but pastures include large areas of floodplain meadows). At the time of our study, it was grazed by one European bison (*Bison bonasus*) and three musk oxen (*Ovibos moschatus*) in an inner fence (50 ha; herbivore density of 3.0 t/km^2^), and approximately 40 Yakutian horses (*Equus ferus*) and several moose (*Alces alces*) in a larger outer fence (1600 ha; herbivore density of 1–2 t/km^2^). Horses were also present in the inner fence from the beginning of the project until about a year prior to our study. Animals are fed in winter to secure their survival and/or build up high-density populations. The “Bisonary” was established in 9 enclosures on 118.5 ha in 2006 and, in 2015, 35 Canadian wood bison (*Bison bison athabascae*; herbivore density of 17.7 t/km^2^) were grazing in mostly secondary meadows, steppes and coniferous taiga. Horses grazed freely on the meadows and steppes outside the fence, except for the steepest slopes.

**Table 1:** Overview on study regions.

Region	Coordinates [WGS 84]	Mean annual temp. [°C]	Annual precipitation [mm]	Main vegetation	Grazing animals
Pokhodsk	69.0667° N 160.9667° E	− 12.9	145	Dwarf shrub and graminoid tundra; tundra steppes	Free roaming reindeer
Chersky	68.7427° N 161.3508° E	− 12.4	155	Larch taiga; floodplain meadows; steppe slopes	"Pleistocene Park" with bison, musk ox, horse, moose; ground squirrels on steppe slopes
Verkhoyansk	67.5506° N 133.3993° E	− 15.4	185	Open larch taiga; floodplain meadows; steppe slopes	Free roaming cattle and horses; ground squirrels
Yakutsk	62.0355° N 129.6755° E	− 9.7	260	Taiga; floodplain meadows; secondary grasslands and steppes	Free roaming cattle and horses; ground squirrels
Buotoma	61.2394° N 128.7649° E	− 9.2	285	Taiga; secondary grasslands and steppes	"Bisonary" with bison; free roaming horses outside the fences

Climate data according to WorldClim^56^.

Livestock (cattle and horses) roamed freely in Verkhoyansk and Yakutsk and wild reindeer (*Rangifer tarandus*) in Pokhodsk; grazing intensity was low to moderate and rather uniform across steppe and tundra-steppe slopes. Some more intensely grazed steppe sites were found around Yakutsk and in the outer areas of the Ust’-Buotoma Bisonary, which was covered with secondary vegetation after clearings of taiga forest and long use as livestock pasture. Small mammals inhabited steppe slopes (mainly ground squirrels, *Urocitellus parryii*).

### Field sampling and data collection

We sampled a total of 210 vegetation plots sized 10 m ×10 m, across steppes and surrounding vegetation types (Table 2). Species were identified in the field and critical taxa in the lab using the “Flora of Siberia”^53^; final taxonomy follows the “Plant List” (http://www.theplantlist.org/). We used the Londo scale to estimate cover of plant species.

**Table 2:** Overview on the number of plots in each single study region per vegetation type.

	Steppe	Meadow	Wetland	Tundra steppe	Tundra	Scrub	Forest	Total
Buotoma	10	16					7	33
Chersky	7		12			20	5	44
Verkhoyansk	41	2				2	15	60
Yakutsk	34	3					3	40
Pokhodsk			7	10	16			33
Total	92	21	19	10	16	22	30	**210**

We measured plant functional traits of the most abundant species in steppes and surrounding vegetation (as suggested by Cornelissen et al.^54^), which depend less on the species turnover across large scales. We chose fundamental traits that are relevant for environmental stress and grazing tolerance or avoidance. Ultimately, we measured 21 traits (Traitset 1 and 2; Appendix S1: Table S1_3; following^54^) for a subset of 92 steppe plots, and 14 traits (Traitset 1; Appendix S1: Table S1_3) for the overall set of plots. The species for which we had measured traits usually covered more than 80% of the biomass of each plot (herb and dwarf shrub layer) and thus should be representative in terms of ecosystem functioning.

We collected biomass of the herb and dwarf shrub layer and soil samples from each vegetation plot in open habitats (meadows, steppes, tundra steppes, shrub openings, forest-steppe ecotones; excluding closed habitats like dwarf shrub tundra, scrub and forest; overall data set of 150 plots). Three subplots with a size of 40 × 40 cm^2^ were randomly selected across the vegetated area of each plot to account for spatial variability. All sampling was carried out in accordance to national and international regulations. In the lab, we measured dry weight (as a proxy for productivity) and nutrient content (%N, %C, C/N ration, Ca, Mg, K and P) of plant biomass, as well as physical and chemical characteristics of soil (fine soil (< 2 mm) fraction, coarse soil (> 2 mm) fraction, pH, electric conductivity (EC), carbonate content, rest water, %N, %C, C/N ratio, Ca, Mg, K and P; see Appendix S1, 1.2 for details).

Slope inclination and aspect were measured in the field. We derived northernness and easternness from aspect (cos/sin), and heat load was calculated according to McCune^55^. Grazing intensity differed between study sites and plots and was approximated based on the density of droppings per plot (dung density in %) by each grazing animal (bison, horse, other large herbivore (mostly cattle, musk ox, moose), small mammals; (for details see Appendix S1, 1.2). Macroclimatic variables (Bio 1/7/10/12/15/18/19) were extracted from WorldClim^56^. For details on environmental variables see Appendix S1, 1.1.

### Data analysis

For the analysis in CANOCO^57^, we square root-transformed species cover, and selected downweighing of rare species in the ordinations. We initially carried out detrended correspondence analysis (DCA) of the entire species set (399 species) for comparison with previous studies (Reinecke et al.^33^). This yielded patterns qualitatively similar to the DCA on the most abundant species only (for which traits were measured), i.e. 217 species in the overall data set and 92 species in the steppe data set. We carried out a principal component analysis (PCA, correlation matrix) to select the most informative and least correlated macroclimatic variables, for further use as co-variables (continentality—Bio 7, winter precipitation—Bio 19, mean summer temperature—Bio10). All explanatory variables were centered and standardized prior to analysis. We carried out analyses for both the overall data set (210 plots for species and traits; 150 plots for biomass) and for a steppe subset (92 plots).

#### Species composition

We first carried out variation partitioning, i.e. serial partial canonical correspondence analysis (pCCA), which tests the unique effects of each group of predictor variables on species composition: macroclimate (continentality, winter precipitation, mean summer temperature), microclimate (slope inclination, heat load, easternness, northernness) and grazing (dung density of bison, horse, other, small mammals). In order to partition out the overriding importance of macrolimate, we then carried out a final pCCA with macroclimate as co-variables and microclimate and grazing as environmental variables, using interactive forward selection to find plot-scale environmental variables which are significant (p < 0.05) in explaining species composition (also see^58^).

#### Plant trait composition

We used plant trait measurements to calculate plot-wise community-weighted means (CWMs) in PC-Ord^59^. Nominal variables were converted to binary dummy variables first, and then weighted by the cover of species per class, and the ordinal variables were standardized. We checked for correlations among CWMs using a correlation matrix PCA. We then carried out a partial redundancy analysis (pRDA) with macroclimate as co-variables and interactive forward selection, analogous to the pCCA described above. We cross-checked the correlation of significant environmental variables with selected CWMs in the ordination using univariate Spearman’s rank correlation.

#### Taxonomic and functional diversity

We differentiated between trait means (CWMs) and trait diversity, because mean traits may be strongly related to productivity and disturbance, but do not necessarily converge within a community^60^. We calculated taxonomic richness and diversity (species richness—S, Shannon index—D and Evenness—E) using the entire species data set of 399 species. For the calculation of functional diversity (Rao’s Q), the respective sets of species with measured traits in the overall and steppe data sets were used. We then used Spearman’s rank correlation to find significant relationships between the diversity indices and the environmental factors (Microclimate and Grazing).

#### Productivity and chemical composition of vegetation

We used the mean of all three subplots to calculate plot-wise soil and biomass data. In a few cases (< 5), data of one subplot was missing and we only used the mean of two subplots. We also analyzed the variation among subplots using the range of measurements within a plot but found no remarkable patterns.

First, we checked for correlations among the plant biomass variables using a correlation-matrix PCA. We then used variation partitioning to assess the importance of the soil and macroclimate variables for biomass variability, as well as the relative contribution of either microclimate or grazing. We first selected the most important soil and macroclimate variables using interactive forward selection in separate RDAs. With these significant soil and macroclimate variables we calculated variation partitioning for three variable groups, separately for both the microclimate and grazing variables. Finally, we carried out a partial redundancy analysis (pRDA) with significant soil (fine soil mass, coarse soil mass, carbonate content, N%, C/N ratio, Mg, P) and macroclimate (summer precipitation, annual temperature, continentality) co-variables and interactive forward selection of the microclimate and grazing variables, analogous to the partial RDA for traits.

## Results

Overall, we found little evidence for soil erosion and overgrazing of steppes, except for narrow trails of grazing animals. In the Bisonary, intensively grazed steppes and meadows had lower vegetation cover and appeared more xeric than the extensively grazed neighboring vegetation. Trampling and wallowing created patches of open and compacted ground. Bison peeled off the bark of trees at their favorite resting places, causing dieback of a whole stand of trees. This effect was much less pronounced in the Pleistocene Park: here the bison opened up the canopy locally by crushing small trees and shrubs, but with little effect on the herb cover.

### Species composition

**Figure 3:**
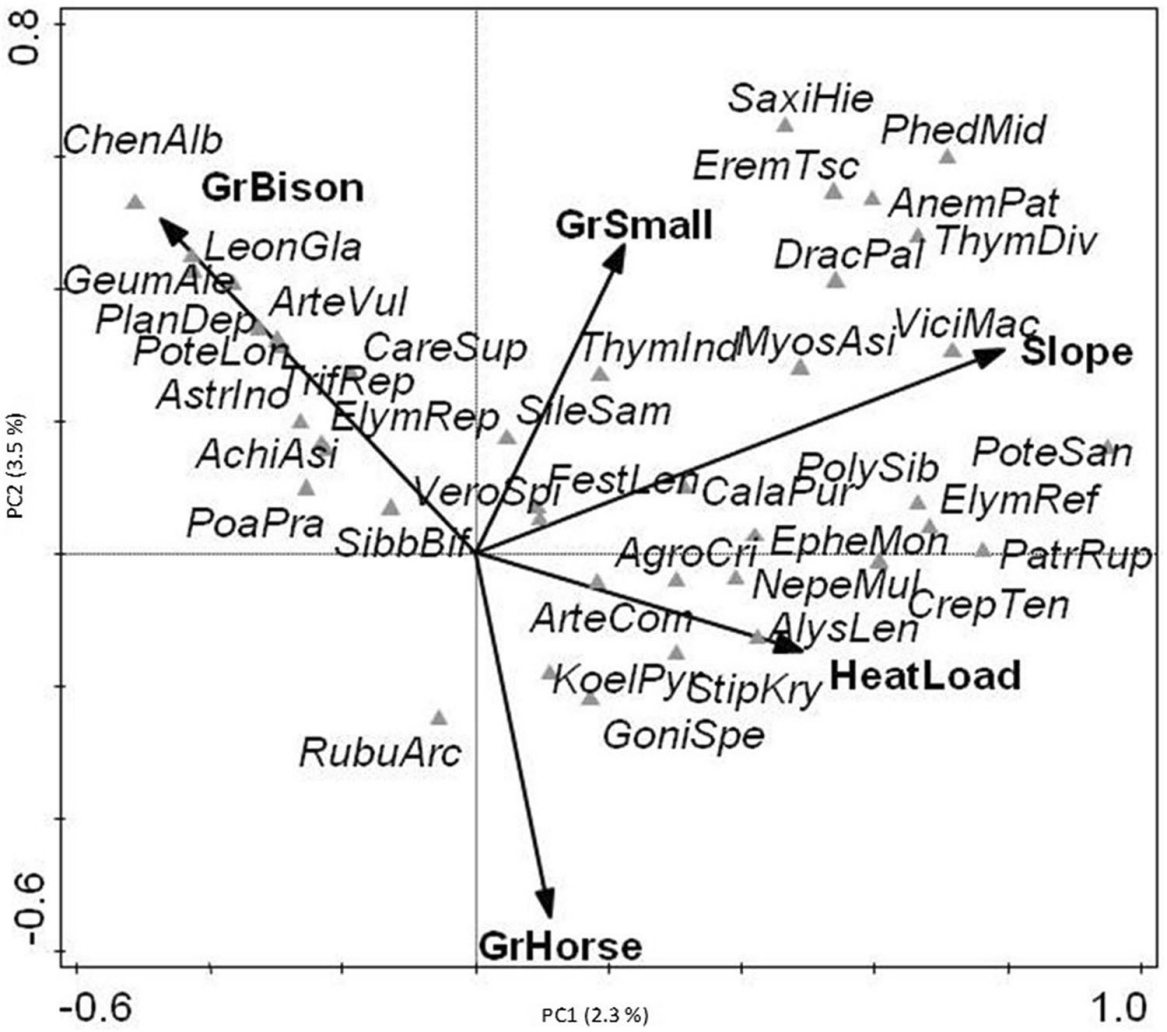
pCCA with macroclimate as co-variable and significant microclimate (slope inclination—Slope; heat load—HeatLoad) and grazing variables (bison—GrBison; horse—GrHorse; small mammals—GrSmall) as constraints (forward selection, 499 permutations); total inertia: 12.9, Eigenvalues: Axis1: 0.29, Axis2: 0.16; %explained variation: Axis1: 2.4, Axis2: 1.3; only most abundant species used and 40 best fitted species shown; species cover square root-transformed.

The DCAs using most abundant species only did not differ substantially from the DCAs using the entire species set; so in the following, we concentrated on the reduced data set. The variation partitioning indicated that macroclimate explains most variance, while microclimate and grazing only explain about half of that each (Table 3). Slope inclination and heat load were the most important microclimatic variables; bison was the most important grazer, followed by horses and small mammals, while other large herbivore-grazing was not found to be significant in the studied locations (Table 3). Dung density tended to be lower with increasing slope inclination (Appendix S1: Figure S1_4), so minor mixed effects are possible. This relationship typically did not vary within study regions though, especially not for the key grazer bison, which evaded the steeper slopes due to lower fodder availability and harder access. After extracting effects of macroclimate, the pCCA (Fig. 3) revealed that *Chenopodium album*, *Leonurus glaucescens*, *Geum aleppicum*, *Plantago depressa*, *Artemisia vulgaris* and *Potentilla longifolia* were related to intensive bison grazing. Other species related to bison grazing were *Trifolium repens* on heavily trampled ground, as well as *Elymus repens* and *Sibbaldianthe bifurca*, which were dominant on drier grazed sites. Indicators for horse grazing were more difficult to discern, but *Goniolimon speciosum* and *Koeleria pyramidata* were correlated with increasing horse grazing. Typical species for small mammal grazing could not be discerned.

**Table 3:** Constrained ordinations of overall data set.

Ordination method	Environmental variable	Variance	% explained
Variation partitioning (pCCAs, unique effects)	Macroclimate	0.52	3.7
	Microclimate	0.24	1.7
	Grazing	0.14	1.0
pCCA (microclimate and grazing |macroclimate)	Slope inclination		1.8
	Grazing bison		1.1
	Grazing horse		0.9
	Heat load		0.8
	Grazing small mammals		0.8

a) Variation partitioning (total inertia: 14.2, total % explained: 10.5%); b) pCCA of microclimate and grazing after removing the effects of macroclimate (total inertia: 12.9, total explained: 5.5%; Eigenvalues: axis 1 = 0.29, axis 2 = 0.16; % explained: axis 1 = 2.3, axis 2 = 1.2). Only significant variables shown.

When considering steppes only, the proportion of variance explained by environmental variables increased (11.9%; Appendix S2: Figure S2_2). Only slope inclination, bison grazing and heatload, as well as easternness were significantly associated with species composition. *Carex supina*, *Chamaerhodos erecta* and *Potentilla longifolia* indicated high bison grazing in steppes (Appendix S2: Figure S2_2). *Linaria acutiloba* and *Sibbaldianthe bifurca* were observed locally with high intensity bison grazing. Horse grazing was less strongly correlated with specific species in steppes, but patterns were similar to those in overall vegetation. *Rubus arcticus* was typical for burnt and more open forest patches, which were often grazed by horses.

### Trait composition

**Figure 4:**
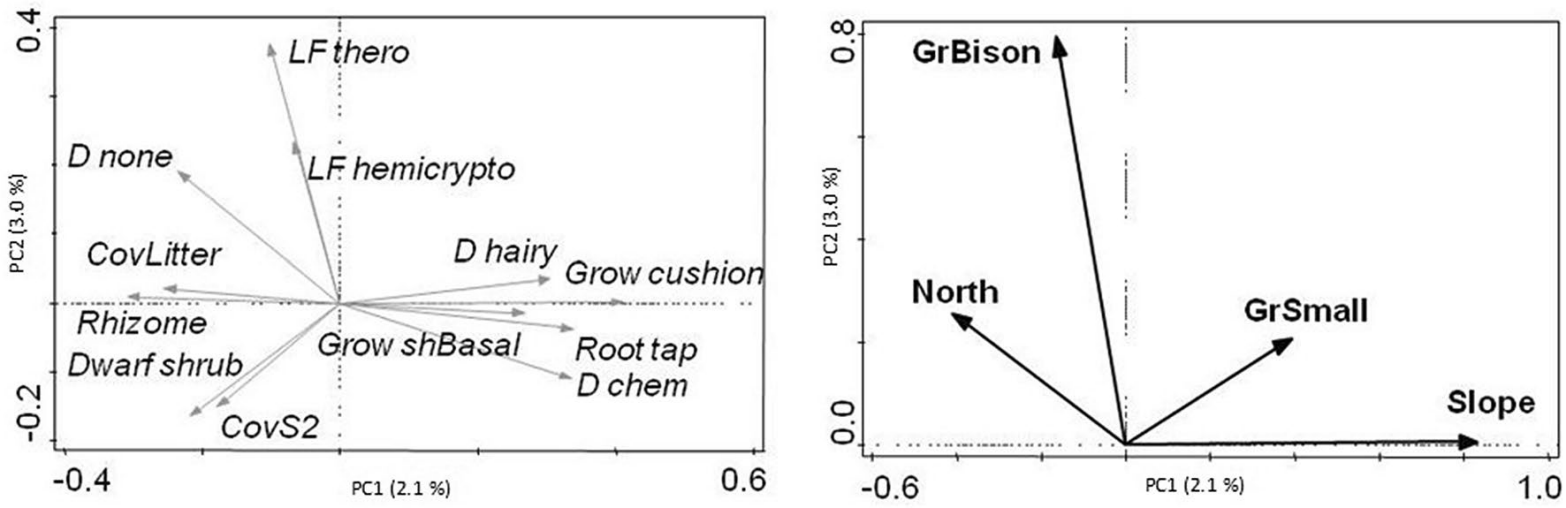
CWM-RDA (forward selection, 499 permutations) of overall data set, total variance: 7604, explained variance: 8.1%, Eigenvalues: Axis1: 0.04, Axis2: 0.01; %explained variation: Axis1: 5.4, Axis2: 1.7; (**a**) CWMs (only 50% most significant traits, which are also confirmed by direct univariate correlation of CWMs with environmental variables, are shown); (**b**) significant microclimate (slope inclination—Slope; northernness—North) and grazing variables (bison—GrBison); small mammals—GrSmall). See Appendix S2: Table S2_1 for abbreviations of plant traits.

In the partial CWM-RDA a total of 8.4% of variation was explained mainly by slope inclination (4.6%) as well as by bison grazing (1.5%), small mammal grazing (1.4%) and northernness (0.8%; significant variables from forward selection only; Fig. 4). Steeper slopes tended to have lower litter cover, but increased numbers of cushions and short-basal plants with taproots/without rhizomes and with chemical defenses. Bison grazing was correlated with an increasing number of hemicryptophytes and therophytes, and a decreasing number of chamaephytes. The number of defenseless plants also increased with bison grazing. Slopes grazed by small mammals featured more cushions, fewer dwarf shrubs (cover and plant functional types) and more hairy plants. Slope northernness was correlated with increased numbers of defenseless, allorhizous plants with rhizomes and decreased proportions of cushions and short-basal plants with taproots and chemical defenses.

When considering steppes only, the partial CWM-RDA explained 10.0% of the total variation, again with slope inclination (5.2%) and bison grazing (2.7%), as well as heat load (2.2%) being the most significant environmental variables (forward selection; Appendix S2: Figure S2_3). The relationship with slope inclination was similar to vegetation overall, though the proportion of stemmy chamaephytes and plants with leathery defenses increased and that of hemicryptophytes decreased. With increasing heat load, the proportion of rosettes decreased, but grasses with closed leaves and plants with large shoot diameter increased. Bison grazing in steppes was correlated with increasing proportions of open ground, as well as a higher proportion of long-leaved plants (leaf length and leaf ratio), while relative inflorescence height tended to decrease.

### Taxonomic and functional diversity

All diversity indices were positively inter-correlated, but indices of taxonomic diversity indices and Rao’s Q were the least correlated variables (Shannon vs. RaoQ: rho = 0.51). In overall vegetation, slope inclination and small mammal grazing were positively, and northernness was negatively correlated with taxonomic and functional diversity indices (except for rho < 0.2 for species richness; Table 4). Heat load and horse grazing were only associated with increasing species richness, heat load also with increasing evenness. Bison grazing was associated with decreased functional diversity, but not with taxonomic diversity. When considering steppes only, the microclimatic or grazing variables had no effect on taxonomic diversity. Functional diversity, however, increased with the easternness of slopes and horse grazing, and decreased with small mammal grazing.

**Table 4:** Correlations of taxonomic and functional diversity with environmental variables.

	Species richness	Shannon index	Evenness	Rao's Q
**(a) Overall**				
Slope inclination	0.21	0.30	0.30	0.45
Heat load	0.36		0.26	
Northernness		−0.22	−0.23	−0.33
Easternness				
Grazing bison				−0.33
Grazing horse	0.24			
Grazing cattle				
Grazing small mammals	0.23	0.36	0.33	0.50
**(b) Steppes**				
Slope inclination				
Heat load				
Northernness				
Easternness				0.23
Grazing bison				
Grazing horse				0.22
Grazing cattle				
Grazing small mammals				−0.23

Spearman correlations of taxonomic (species richness, Shannon, Evenness) and functional (Rao’s Q) diversity indices with environmental variables for microclimate and grazing impact for a) the overall data set, as well as b) separately for steppes. Spearman’s rho, only values > 0.2 are given.

### Productivity and chemical composition of vegetation

The PCA revealed that biomass characteristics are rather divergent and generally reflect differences in soil conditions between steppes and overall vegetation (Appendix S2: Figure S2_4). According to variation partitioning, biomass characteristics were mainly explained by soil conditions and macroclimate, both overall (total explained: 48.7%) and in steppes (total explained: 34.8%). Microclimate (overall: 1.8%; steppes: 1.4%) and grazing (overall: 1.6%; steppes: 1.3%) added little additional explanation (Appendix S2: Table S2_2).

**Figure 5:**
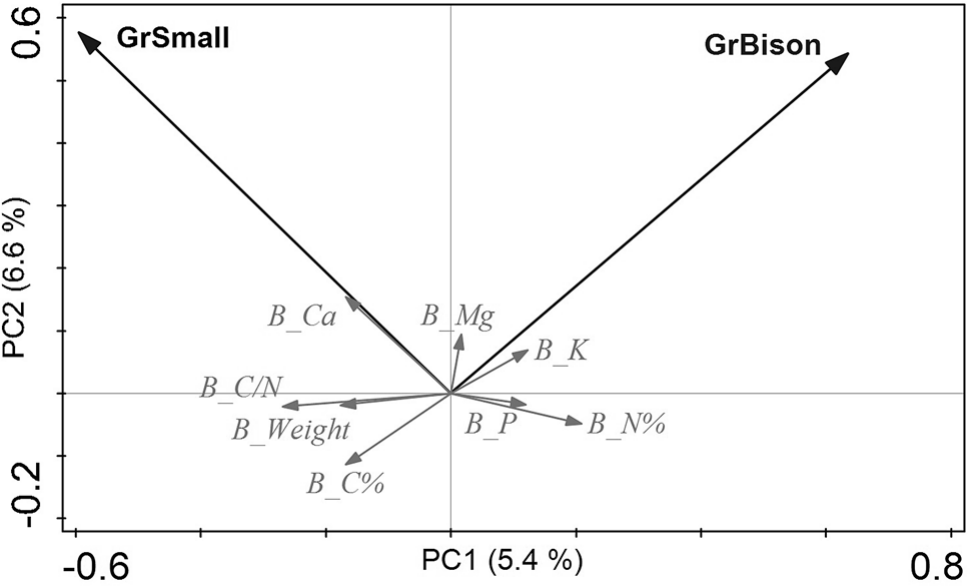
Biomass-RDA (forward selection, 499 permutations) of the open habitat data set, total variance: 652, explained variance: 6.6%, Eigenvalues: Axis1: 0.03, Axis2: 0.01; %explained variation: Axis1: 5.4, Axis2: 6.6; only significant grazing variables (bison—GrBison); small mammals—GrSmall) are shown.

In the partial RDA of overall biomass data, only a total of 6.6% of remaining variation were explained, mainly by bison grazing (3.6%) and small mammal grazing (3.0%; significant variables from forward selection only; Fig. 5). Bison grazing was correlated with lower weights of harvested biomass, lower C content and C/N ratio, but increased Mg and K concentrations. Small mammal grazing was related to increased Ca, but lower N and P concentrations.

When considering steppes only, the partial RDA with macroclimate as covariable explained a total of 3.0% of biomass variance (Appendix S2: Figure S2_5). Heat load was the only significant variable from forward selection (3.0%) and was slightly correlated with increased productivity and nutrient content (P, N%, K). Grazing was not important in explaining biomass variance in steppes.

## Discussion

Our data suggests that standard rewilding theories based on top-down controls by large herbivores may not be applicable in the context of cold steppe outposts, or at least that Pleistocene conditions of the mammoth steppe are not easily restored today. The harsh continental to suboceanic climate gradient was the overriding factor for species composition of current vegetation in our study area. It determines the zonal distribution of tundra in the north, open larch taiga in the centre of the study region and coniferous taiga south of Yakutsk (e.g.^61^), which explains the high species turnover across our data set. Topography and microclimate determine the occurrence of extrazonal steppes^32^ and azonal vegetation like floodplain meadows, yet even within these habitat types, large-scale climatic gradients had an influence as shown by analyses on steppe data only. Trait assemblages changed less than species assemblages across the study area, supporting the hypothesis that functional composition remains more constant along environmental gradients (see^54^). The productivity of vegetation, especially of steppe vegetation, was also more a result of climate and soil conditions, than influenced by grazing.

### Effect of microclimate

Within our dataset, microclimate was slightly more important than grazing for explaining the species composition overall and specifically of steppe vegetation. Slope inclination determines water run-off as well as the degree of stress by soil movement. Petrophytic and xeric extrazonal steppes are thus confined to steep slopes with southwestern exposure and high heat load^32^. These steppes feature a high share of cushions, grasses with closed leaves, as well as broad, stemmy chamaephytes (*Artemisia* species) and plants with leathery leaves, while the proportion of semi-basal hemicryptophytes is low. Thus, steppes are not only hotspots of taxonomic diversity^62^ but also of functional diversity in the generally rather species poor taiga and tundra zones. Microclimate seemed to be less important around Yakutsk, were steppes could be found on a wider range of slope inclinations and even in plains. There, long-term livestock grazing may have locally extended the otherwise topographically determined distribution of steppes.

### Grazing effects

The effects of large herbivores on the studied vegetation were rather small, both across the study regions and on the local scale. The magnitude of change depended on the vegetation type, grazer species, grazing intensity, habitat use and on interactions of grazing with climate and soil conditions.

Among the variables studied, steppe vegetation was least affected by herbivores. This may reflect co-adaptation to special microclimatic conditions^63,64^. Hairiness, taproots, cushion growth form, bunch-grasses and chemical compounds which characterize drought-adapted steppe plants are also suitable traits to tolerate or avoid grazing^63,64^. According to general models, grazing avoidance should be favored against grazing tolerance in situations of water or nutrient scarcity^65,66^. The fast growing grasses of floodplain meadows, on the other hand, are adapted to re-growth after flooding and also should be tolerant to moderate grazing disturbance^47,67^. Thus, shifts in species and trait composition were only visible in vegetation types where grazing affected plant resources: in meadows, where trampling and soil compaction created drier and more disturbed soil conditions; and in forest clearings and meadows, where droppings and urine deposition increased nutrient availability at resting places. Steppe plant biomass was also mainly characterized by climate and soil conditions and grazing had insignificant influence on the productivity and nutritious quality of forage.

Overall, our study could not detect relevant effects on most of the studied vegetation. We could not properly assess the importance of reindeer grazing because it is restricted to winter periods in the Yana and Kolyma regions, so finding evidence for grazing and trampling was difficult. The observed soil disturbances at the tundra steppe sites could originate from both frost and wind erosion and/or trampling disturbances (see also^32^). Moreover, small mammals have been proposed as ecosystem engineers in other steppe environments (e.g.^68^). In Yakutia, the presence of small mammals like the arctic ground squirrel (*Urocitellus parryi*) is probably mostly related to the openness of steppe slopes offering appropriate habitats and an active layer sufficiently drained for digging dens. However, steppe rodent activity might be more important for plant community composition on the local scale. Surprisingly, ground squirrels were related to declining functional diversity in steppes. The often positive effect of their burrowing activities on plant diversity^69,70^ might be less important on steppe slopes featuring constant soil erosion, while selective feeding could actually decrease diversity.

The dominant large herbivores, bison and horses, exerted small, yet significant grazing pressure. Bison had the largest and most consistent effects on species and trait composition of steppe and surrounding vegetation. Debarking of trees locally opened up forest patches, and urine deposition fertilized the soil, creating denser vegetation with ruderal plant species (e.g. *Geum aleppicum*, *Artemisia vulgaris*). Trampling and wallowing created patches of bare soil allowing the establishment of indicators for disturbance and soil compaction. The occurrence of drought-adapted species can be interpreted as slight xerophytization of meadows through bison grazing. Intensive bison grazing is also related to a high share of hemicryptophytic grasses, the animals’ preferred food over herbs^71^. The lower harvested biomass weight indicates heavy plant removal by bison, while fertilization and possibly re-allocation increased concentration of nitrogen and other nutrients in the remaining plant biomass.

We could discern two indicators for horse grazing (*Goniolimon speciosum*, *Koeleria pyramidata*), which are typical for moderately grazed extrazonal steppes in general^72–74^. A typical indicator for heavy grazing in Central Yakutia, *Carex duriuscula*^49,75^, could not be confirmed for our study locations. Indicator species found are generally characteristic for Yakutian steppes (e.g.^32^), but their high abundances can be interpreted as consequences of moderate grazing of all studied steppes. Sergey Zimov’s (personal communication) and our field observation of reduced litter cover and a shift from tussocks to rhizomatous grasses in grazed floodplain meadows, might be an important local effect, but could not be confirmed or generalized across our study areas.

The population density of large herbivores, especially of bison differed strongly between the two studied enclosures: while the effects were obvious in the Bisonary (with over 30 bison), little change could be observed in the Pleistocene Park (with only one bison and three musk oxen). This might be related to the lower productivity of pastures (Appendix S1: Figure S1_2) as well as less suitable habitats for grazing (Table 1) in the Pleistocene Park. The grazer’s habitat use also influences relationships between grazing intensity and plant traits. Bison grazing was related to reduced functional diversity. Large-leaved productive grasses and other hemicryptophytes without defense mechanisms dominated the meadows in the plains, which bison preferably used as pastures. Herbs, shrubs and dwarf shrubs with more variable traits were found in less productive and less grazed vegetation. Thus, our data does not reveal whether grazing actually promotes a higher proportion of grasses in the vegetation, or whether grazing intensity is high there because of the grassland’s high forage quality. Similarly, the observed positive relationship between horse grazing and respective indicator species, species richness and functional diversity in steppes is probably also an effect of habitat use. Horse grazing has a long tradition in Yakutia^50,76^, and despite low grazing densities, steppes are regularly used. Thus, these results indicate the regular but moderate use of all steppes by freely roaming horses. On the other hand, grazers need to be able to track changes in forage quantity and quality^77,78^. Fencing prevented free migration of bison, and supplementary feeding in winter helps to keep population densities high, resulting in uncoupled grazing from vegetation and creating a risk of overgrazing in summer.

The potential of grazing effects depends strongly on climate and soil conditions. In the suboceanic climate of the Pleistocene Park under seasonal inundation and without any steppe vegetation within the vicinity, abiotic constraints did not allow for lasting grazing effects. Large herbivores concentrated on floodplain meadows, which are only accessible after drainage and drying up in mid-to late summer, and when boggy soils are frozen in winter. Trampling and compaction of snow decreases insulation and thus temperature of the soil, which in turn affects permafrost soil conditions (N. Zimov, unpublished data). In these areas, grazing has reduced the litter cover and the size and proportion of tussock grasses over time (S. Zimov, personal communication), and in comparison to floodplain meadows outside the fence. Horses were also attracted to forest and scrub openings, induced by fire and logging, where soil disturbance as a result of altered permafrost conditions facilitated development of an increased herb cover. Effects of bison were much more pronounced in the Bisonary, where they extensively peeled off the bark of trees at their favorite resting places, thus leading to the dieback of a whole stand of trees.

In the fully continental climate of Central Yakutia, net precipitation (precipitation minus evapotranspiration) is closer to the aridity threshold than in the more oceanic Kolyma region. Even small grazing-induced shifts in the moisture regime (decreased shading, removal of insulating mosses, higher water run-off from compacted soil) can create substantially more arid conditions. In effect, bison grazing in the Bisonary led to the xerophytization of meadows and long-term grazing of livestock around Yakutsk probably enlarged the area of steppes, whose occurrence is otherwise determined by dry microclimate.

Due to the lack of means by which to assess herbivore densities outside the two rewilding sites it could still be possible that grazing intensity there was too low to find relevant grazing effects. In principle, free grazing should be more similar to natural conditions than any fence-based management, yet renders estimation of grazing intensities difficult. This is a common problem in rangeland ecology whenever it comes to open range or even mobile grazing systems, such as in the steppes of Mongolia. Livestock grazing there concentrates around water wells and grazing intensity constantly decreases towards the outer perimeter^79,80^. This was not the case in Yakutia. In search for fodder in a heterogenous landscape, livestock roams around between pastures, and grazing is not focused around a central point, since water is readily available. Herbivore density of free roaming livestock would be somewhere between that of the Bisonary (where it is highest with 17.7 t/km^2^) and the large enclosure of the Pleistocene Park (where it is lowest with 1.0 t/km^2^) in the larger vicinity of villages. The fact that Zimov’s animals have difficulties surviving the harsh winters despite receiving additional fodder supports the notion that current herbivore densities in our focal sites at least are probably not much lower than carrying capacity.

### Implications for rewilding in Siberia

Field studies, such as the present one, often lack proper control treatments (e.g.^47^). Due to the grazer’s selective habitat use, we can only claim that grazing and productive grasslands are associated, not whether grazing promotes this type of vegetation. The palatability of plant species is relative, not absolute—whether they are eaten or not depends on what else is available^81^. We can, however, attribute the occurrence of plant species related to disturbance and soil compaction and probably the restriction of shrub encroachment to the activities of large herbivores.

Our study supports the notion that local context (climate, soils, vegetation type, grazing animals) is important for effects of rewilding on vegetation and generalizations might be difficult even if a more robust trait-based approach is pursued^81^.

First, there is evidence that direction and magnitude of vegetation change strongly depend on the type of herbivore (e.g. Suominen and Olofsson^43^). Domestic horses and cattle are almost exclusive grazers^82^, while wood bison supplement their grazing diet with browsing and bark stripping^83^. Bison were not only larger than livestock, but behavior like wallowing, bark stripping and crushing shrubs, which seemed to be more important than actual grazing, was also restricted to bison. Bison were among the most abundant large herbivores in northern Siberia during Pleistocene cold stages, together with mammoth, horses and reindeer^14,21,84,85^. Due to their destructive foraging behavior, mammoth and woolly rhino have been proposed as keystone species for sustaining openings and small grass communities in forests^10,27,86^. In this regard bison might be the most effective of extant northern large herbivores in the ability to alter habitats^6^ and thus functionally closest to the forage type of Proboscideans like the mammoth^3,19,87,88^. Yet the critical impact on vegetation possibly might be hardly reached with this extant herbivore species (also see^52^) and the attempt to replace unique feeding niches by artificial forest clearing or high densities of other grazers alone^14^ seems to be insufficient. The mammoth is, after all, the most dominant and important keystone species in many fossil bone assemblages^21^, with no modern equivalent in livestock or any other large herbivore of the boreal zone.

Second, studies have shown that multi-species assemblages of at least two different species of herbivores are essential to trigger lasting changes in vegetation^44,89–91^. Even relatively closely related grazers, such as bison and cattle, can have very different grazing habits and resulting impacts on their pastures^71^. In both the Pleistocene Park and the Bisonary, horses and bison had originally been grazed together, but due to strong aggressive behavior from bison towards the horses, they had to be separated. Unlike in a natural environment, where grazers could evade contact, yet forage in the same habitats at different times, possible synergistic effects were excluded. Local herbivore communities in the mammoth steppe were obviously more diverse than they are in our experimental settings, with a dominance of woolly mammoth (25–35%), followed by horse (20–25%), bison (15–20%) and reindeer (14–18%) and a smaller contribution of other large herbivores, such as musk ox, woolly rhino and saiga antelope^21^. In modern herbivore assemblages, woolly mammoth, woolly rhino and saiga have no modern analogues, musk ox and re-introduced bison only play a local role, and horse and reindeer have largely been replaced by domesticated forms. This difference in herbivore diversity might be a key factor for the low grazing effects observed (also see^52^).

And third, the re-conversion of tundra and taiga into mammoth steppe most probably requires different processes than those needed for mere maintenance of an existing ecosystem. Tundra and taiga ecosystems might be so inherently different in all their characteristics that a tipping point might have been reached, which does not allow for a simple re-conversion^92,93^. However, this does not mean that these ecosystems are invariant to transformations by large herbivores. We conclude that the aim to bring back the lost mammoth steppe ecosystem seems beyond reach in the near future, but positive effects of rewilding in creating landscape mosaics of heterogeneous plant communities are promising conservation and research aims and should be carefully studied in the future.

## Supplementary Information

Supplementary Information 1.

## Data Availability

Data on grassland vegetation has been contributed to the Database of Scale-Dependent Phytodiversity Patterns in Palaearctic Grasslands (GrassPlot).

## Abbreviations

AchiAsi: Achillea asiatica
AgroCri: Agropyron cristatum
AgroVin: Agrostis vinealis
AlysLen: Alyssum lenense
AlysObo: Alyssum obovatum
AnemPat: Anemone patens
ArteCom: Artemisia commutata
ArteSan: Artemisia santolinifolia
ArteTan: Artemisia tanacetifolia
ArteVul: Artemisia vulgaris
AstrIno: Astragalus inopinatus
CalaPur: Calamagrostis purpurascens
CareDur: Carex duriuscula
CarePed: Carex pediformis
CareSup: Carex supina
ChamEre: Chamaerhodos erecta
ChenAlb: Chenopodium album
CrepTen: Crepidifolium tenuifolium
DracPal: Dracocephalum palmatum
ElymRef: Elymus reflexiaristatus
ElymRep: Elymus repens
EpheMon: Ephedra monosperma
EremTsc: Eremogone tschuktschorum
EuphEsu: Euphorbia esula
FestLen: Festuca lenensis
GaliVer: Galium verum
GeumAle: Geum aleppicum
GoniSpe: Goniolimon speciosum
HeliHoo: Helictotrichon hookeri
KoelPyr: Koeleria pyramidata
LeonGla: Leonurus glaucescens
LinaAcu: Linaria acutiloba
MyosAsi: Myosotis asiatica
NepeMul: Nepeta multifida
PatrRup: Patrinia rupestris
PhedMid: Phedimus middendorffianus
PlanDep: Plantago depressa
PoaPra: Poa pratensis
PolySib: Polygala sibirica
PoteAre: Potentilla arenosa
PoteLon: Potentilla longifolia
PoteSan: Potentilla sanguisorba
PoteTol: Potentilla tollii
RubuArc: Rubus arcticus
SaxiHie: Saxifraga hieraciifolia
SelaSel: Selaginella sellowii
SibbBif: Sibbaldianthe bifurca
SileSam: Silene samojedorum
SpirMed: Spiraea media
StelLon: Stellaria longipes
StipKry: Stipa krylovii
ThymDiv: Thymus diversifolius
ThymInd: Thymus indigirkensis
TrifRep: Trifolium repens
VeroSpi: Veronica spicata
ViciMac: Vicia macrantha

## References

[1] Doughty CE, Wolf A, Field CB (2010). Biophysical feedbacks between the Pleistocene megafauna extinction and climate: The first human-induced global warming?. Geophys. Res. Lett..

[2] Svenning J-C (2016). Science for a wilder Anthropocene: Synthesis and future directions for trophic rewilding research. Proc. Natl. Acad. Sci..

[3] Owen-Smith N (1987). The pivotal role of megaherbivores. Paleobiology.

[4] Vera FWM (2000). Grazing Ecology and Forest History.

[5] Zimov SA (1995). Steppe-Tundra transition: A herbivore-driven biome shift at the end of the pleistocene. Am. Nat..

[6] Gill JL (2014). Ecological impacts of the late quaternary megaherbivore extinctions. New Phytol..

[7] Bakker ES (2016). Combining paleo-data and modern exclosure experiments to assess the impact of megafauna extinctions on woody vegetation. Proc. Natl. Acad. Sci..

[8] Martin PS, Wright HE (1967). Pleistocene Extinctions: The Search for a Cause, Vol 6***.

[9] Haynes G, DellaSala D, Goldstein M (2018). The evidence for human agency in the late Pleistocene megafaunal extinctions. Encyclopedia of the Anthropocene, voxl 1.

[10] Johnson CN (2009). Ecological consequences of Late Quaternary extinctions of megafauna. Proc. R. Soc. B Biol. Sci..

[11] Gradmann R (1933). Die Steppenheidentheorie. Geogr. Z..

[12] Pausas JG, Bond WJ (2020). Alternative biome states in terrestrial ecosystems. Trends Plant Sci..

[13] Zimov SA, Zimov NS, Tikhonov AN, Chapin FS (2012). Mammoth steppe: A high-productivity phenomenon. Quat. Sci. Rev..

[14] Zimov SA, Zimov NS, Chapin FS (2012). The past and future of the mammoth steppe ecosystem. Springer Earth Syst. Sci..

[15] Zimov SA (2005). Pleistocene park: Return of the Mammoth’ s ecosystem. Science (80–).

[16] Yurtsev BA (2001). The pleistocene ‘Tundra-steppe’ and the productivity paradox: The landscape approach. Quat. Sci. Rev..

[17] Blinnikov MS, Gaglioti BV, Walker DA, Wooller MJ, Zazula GD (2011). Pleistocene graminoid-dominated ecosystems in the Arctic. Quat. Sci. Rev..

[18] Kienast F, Elias SA, Mock C (2013). Plant macrofossil records—Arctic Eurasia. Encyclopedia of Quaternary Science.

[19] Guthrie RD, Hopkins DM, Matthews JV, Schweger CE, Young SB (1982). Mammals of the mammoth steppe as paleoenvironmental indicators. Paleoecology of Beringia.

[20] Kienast F, Schirrmeister L, Siegert C, Tarasov P (2005). Palaeobotanical evidence for warm summers in the East Siberian Arctic during the last cold stage. Quat. Res..

[21] Sher AV, Kuzmina SA, Kuznetsova TV, Sulerzhitsky LD (2005). New insights into the Weichselian environment and climate of the East Siberian Arctic, derived from fossil insects, plants, and mammals. Quat. Sci. Rev..

[22] Guthrie RD (2001). Origin and causes of the mammoth steppe: A story of cloud cover, woolly mammal tooth pits, buckles, and inside-out Beringia. Quatern. Sci. Rev..

[23] Rivals F, Semprebon G, Lister A (2012). An examination of dietary diversity patterns in Pleistocene proboscideans (Mammuthus, Palaeoloxodon, and Mammut) from Europe and North America as revealed by dental microwear. Quat. Int..

[24] van Asperen EN, Kahlke R-D (2017). Dietary traits of the late Early Pleistocene Bison menneri (Bovidae, Mammalia) from its type site Untermassfeld (Central Germany) and the problem of Pleistocene ‘wood bison’. Quat. Sci. Rev..

[25] Saarinen J, Lister AM (2016). Dental mesowear reflects local vegetation and niche separation in Pleistocene proboscideans from Britain. J. Quat. Sci..

[26] Sher AV (1968). Fossil saiga in northeastern Siberia and Alaska. Int. Geol. Rev..

[27] Kahlke RD, Lacombat F (2008). The earliest immigration of woolly rhinoceros (Coelodonta tologoijensis, Rhinocerotidae, Mammalia) into Europe and its adaptive evolution in Palaearctic cold stage mammal faunas. Quat. Sci. Rev..

[28] Kahlke RD (2014). The origin of Eurasian Mammoth Faunas (Mammuthus-Coelodonta Faunal Complex). Quat. Sci. Rev..

[29] Rivals F, Lister AM (2016). Dietary flexibility and niche partitioning of large herbivores through the Pleistocene of Britain. Quat. Sci. Rev..

[30] Kahlke RD (2015). The maximum geographic extension of Late Pleistocene Mammuthus primigenius (Proboscidea, Mammalia) and its limiting factors. Quat. Int..

[31] Chapin FS, Shaver RR, Giblin AE, Nadelhoffer KG, Laundre JA (1995). Response of arctic tundra to experimental and observed changes in climat. Ecology.

[32] Reinecke J, Troeva E, Wesche K (2017). Extrazonal steppes and other temperate grasslands of northern Siberia—phytosociological classification and ecological characterization. Phytocoenologia.

[33] Yurtsev BA, Hopkins DM, Matthews JV, Schweger CE, Young SB (1982). Relics of the xerophyte vegetation of Beringia in northeastern Asia. Paleoecology of Beringia.

[34] Ashastina K (2018). Woodlands and steppes: Pleistocene vegetation in Yakutia’s most continental part recorded in the Batagay permafrost sequence. Quartern. Sci. Rev..

[35] Chytrý M (2017). Refugial ecosystems in central Asia as indicators of biodiversity change during the Pleistocene–Holocene transition. Ecol. Indic..

[36] Gill JL, Williams JW, Jackson ST, Lininger KB, Robinson GS (2009). Pleistocene megafaunal collapse, novel plant communities, and enhanced fire regimes in North America. Science (80–).

[37] Cingolani AM, Noy-Meir I, Díaz S (2005). Grazing effects on rangeland diversity: A synthesis of contemporary models. Ecol. Appl..

[38] Wehrden HV, Hanspach J, Kaczensky P, Fischer J, Wesche K (2012). Global assessment of the non-equilibrium concept in rangelands. Ecol. Appl..

[39] Wang Y (2017). Combined effects of livestock grazing and abiotic environment on vegetation and soils of grasslands across Tibet. Appl. Veg. Sci..

[40] Elser JJ (2007). Global analysis of nitrogen and phosphorus limitation of primary producers in freshwater, marine and terrestrial ecosystems. Ecol. Lett..

[41] Manseau M, Huot J, Crête M (1996). Effects of summer grazing by caribou on composition and productivity of vegetation: Community and landscape level. J. Ecol..

[42] Suominen O, Olofsson J (2000). Impacts of semi-domesticated reindeer on structure of tundra and forest communities in fennoscandia: A review. Ann. Zool. Fennici.

[43] Virtanen R (2000). Effects of grazing on above-ground biomass on a mountain snowbed, NW Finland. Oikos.

[44] Ravolainen VT (2011). Rapid, landscape scale responses in riparian tundra vegetation to exclusion of small and large mammalian herbivores. Basic Appl. Ecol..

[45] Wang Y, Wesche K (2016). Vegetation and soil responses to livestock grazing in Central Asian grasslands: A review of Chinese literature. Biodivers. Conserv..

[46] Díaz S, Noy-meir I, Cabido M (2001). Can grazing of herbaceous plants be predicted response from simple vegetative traits?. J. Appl. Ecol..

[47] Díaz S (2007). Plant trait responses to grazing—a global synthesis. Glob. Change Biol..

[48] Pakeman RJ, Marriott CA (2010). A functional assessment of the response of grassland vegetation to reduced grazing and abandonment. J. Veg. Sci..

[49] Troeva EI, Cherosov MM (2012). Transformation of Steppe communities of Yakutia due to climatic change and anthropogenic impact in Eurasian Steppes. Ecol. Probl. Livelih. Changing World.

[50] Gavrilyeva L, Sofronov R, Arzhakova A, Barashkova N, Ivanov I, Al T (2010). Hayfields and pastures. The Far North: Plant Biodiversity and Ecology of Yakutia.

[51] Gill JL (2015). Learning from Africa’s herbivores. Science (80–).

[52] Reinecke JSF (2019). The Return of the Mammoth Steppe?—Rewilding in Yakutia and the Actual Impact of Large Herbivore Grazing on Vegetation.

[53] Malyschev LI (2006). Flora of Siberia.

[54] Cornelissen JHC (2003). A handbook of protocols for standardised and easy measurement of plant functional traits worldwide. Aust. J. Bot..

[55] McCune B (2007). Improved estimates of incident radiation and heat load using non-parametric regression against topographic variables. J. Veg. Sci..

[56] Hijmans RJ, Cameron SE, Parra JL, Jones PG, Jarvis A (2005). Very high resolution interpolated climate surfaces for global land areas. Int. J. Climatol..

[57] Ter Braak, C. J. F. & Šmilauer, P. Canoco reference manual and user’s guide: Software for ordination. 496 (2012).

[58] Ashastina K (2018). Palaeo-environments at the Batagay site in West Beringia During the Late Quaternary.

[59] McCune, B. & Mefford, M. J. PC-ORD. (2011).

[60] Pakeman RJ, Lennon JJ, Brooker RW (2011). Trait assembly in plant assemblages and its modulation by productivity and disturbance. Oecologia.

[61] Troeva EI, Isaev AP, Cherosov MM, Karpov NS (2010). The Far North: Plant Diversity and Ecology of Yakutia.

[62] Elvebakk A (2005). ‘Arctic hotspot complexes’—proposed priority sites for studying and monitoring effects of climatic change on arctic biodiversity. Phytocoenologia.

[63] Coughenour MB (1985). Graminoid responses to grazing by large herbivores: Adaptations, exaptations, and interacting processes. Ann. Missouri Bot. Gard..

[64] Quiroga RE, Golluscio RA, Blanco LJ, Fernández RJ (2010). Aridity and grazing as convergent selective forces: An experiment with an Arid Chaco bunchgrass. Ecol. Appl..

[65] Herms DA, Matson WJ (1992). The dilemma of plants: To grow or defend. Q. Rev. Biol..

[66] Hobbie SE (1992). Effect of plant species on nutrient cycling. Trends Ecol. Evol..

[67] Coley PD, Bryant JP, Chapin FS (1985). Resource availability and plant antiherbivore defense. Science (80–).

[68] Wesche K, Nadrowski K, Retzer V (2007). Habitat engineering under dry conditions: The impact of pikas (*Ochotona pallasi*) on vegetation and site conditions in southern Mongolian steppes. J. Veg. Sci..

[69] Newediuk LJ, Waters I, Hare JF (2015). Aspen parkland pasture altered by Richardson’s ground squirrel (*Urocitellus richardsonii* Sabine) activity: The good, the bad, and the not so ugly?. Can. Field-Nat..

[70] Wheeler HC, Hik DS (2013). Arctic ground squirrels *Urocitellus parryii* as drivers and indicators of change in northern ecosystems. Mamm. Rev..

[71] Steuter AA, Hidinger L (1999). Comparative ecology of bison and cattle on mixed-grass prairie. Gt. Plains Res..

[72] Ivanova, V. Tipchakovye stepi—odin iz etapov pastbischnoi digressii rastitelnosti v doline srednei Leny. In *Rastitelnost Yakutii i Eyo Okhrana* (ed. Andreyev, V.) 37–56 (1981).

[73] Ivanova, V. O vliyanii vypasa na stepnuyu rastitelnost v doline r. Leny. In *Lyubite i okhranyaite prirodu Yakutii* 86–93 (1967).

[74] Gavrilyeva L (1998). Pastbishnaya Digressiya i Ratsionalnoye Ispolzovaniye Rastitelnosti Alasov Leno-Amginskogo Mezhdurechya.

[75] Bazha SN, Gunin PD, Danzhalova EV, Drobyshev YI, Prishcepa AV, Werger MJA, Staalduinen MA (2012). Pastoral degradataion of steppe ecosystems in Central Mongolia. Eurasian Steppes. Ecological Problems and Livelihoods in a Changing World.

[76] Crate S (2017). Permafrost livelihoods: A transdisciplinary review and analysis of thermokarst-based systems of indigenous land use. Anthropocene.

[77] Ellis J, Swift D (1988). Stability of African pastoral ecosystems: Alternate paradigms and implications for development. J. Range Manag..

[78] Nachinshonhor UG, Yamamura N, Fujita N, Maekawa A (2014). Use of steppe vegetation by nomadic pastoralism in Mongolia. Ecological Research Monographs.

[79] Wang Y (2018). Multiple indicators yield diverging results on grazing degradation and climate controls across Tibetan pastures. Ecol. Indic..

[80] Ahlborn J (2020). Climate—grazing interactions in Mongolian rangelands: Effects of grazing change along a large-scale environmental gradient. J. Arid Environ..

[81] Vesk PA, Westoby M (2001). Predicting plant species’ responses to grazing. J. Appl. Ecol..

[82] Shipley, L. Grazers and browsers: how digestive morphology affects diet selection. *Grazing behavior of livestock and wildlife* **70**, 20–27 (1999).

[83] Larter, N. C. Diet and habitat selection of an erupting wood bison population. 1–118 (1988).

[84] Kuznetsova, T. V. *Fossils of the mammoth fauna*. *Russian-German Cooperation SYSTEM LAPTEV SEA: The Expedition Lena—New Siberian Islands 2007 during the International Polar Year 2007/2008,* 139–140 (2008).

[85] Kuznetsova, T. V., Sulerzhitsky, L. D. & Siegert, C. New data on the ‘Mammoth’ fauna of the Laptev Shelf Land (East Siberian Arctic). In *The World of Elephants—International Congress* 289–292 (2001).

[86] Haynes G (2012). Elephants (and extinct relatives) as earth-movers and ecosystem engineers. Geomorphology.

[87] Gill R, Danell K, Bergström R, Duncan P, Pastor J (2006). The influence of large herbivores on tree recruitment and forest dynamics. Large Herbivore Ecology, Ecosystem Dynamics and Conservation.

[88] Martin PJ, Hopkins DM, Matthews JV, Schweger CE, Young SB (1982). Digestive and grazing strategies of animals in the arctic steppe. Paleoecology of Beringia.

[89] Huisman J, Olff H (1998). Competition and facilitation in multispecies plant-herbivore systems of productive environments. Ecol. Lett..

[90] Waldram MS, Bond WJ, Stock WD (2008). Ecological engineering by a mega-grazer: White Rhino impacts on a south African savanna. Ecosystems.

[91] Cornelissen P (2017). Large Herbivores as a Driving Force of Woodland-Grassland Cycles.

[92] Scheffer M, Carpenter SR (2003). Catastrophic regime shifts in ecosystems: Linking theory to observation. Biotechnol. Agron. Soc. Environ..

[93] Scheffer M, Hirota M, Holmgren M, Van Nes EH, Chapin FS (2012). Thresholds for boreal biome transitions. Proc. Natl. Acad. Sci..

