# Supplementary material for: Effects of large herbivore grazing on relics of the presumed mammoth steppe in the extreme climate of NE-Siberia
Source: Sci Rep. 2021 Jun 21;11:12962. doi: 10.1038/s41598-021-92079-1 (PMC8217226; doi:10.1038/s41598-021-92079-1)
Supplement: Supplementary file 1 — Supplementary Information 1. [file 41598_2021_92079_MOESM1_ESM.pdf]

Supplementary information

Effects of large herbivore grazing on relics of the presumed mammoth steppe in the extreme climate of NE-Siberia

J. Reinecke, K. Ashastina, F. Kienast, E. Troeva, K. Wesche

Submitted on
27.1.2021

Appendix S1: Supplementary information for introduction and details on methods

Table S1_1. List of known mammal species of the Late Pleistocene mammoth steppe biome in northeastern Eurasia and their current status in the region.

Species group	Species		Current status	References
Large herbivores				
	Mammuthus primigenius	Woolly Mammoth	Extinct; no modern analogue	Boeskorov 2004; Kahlke 2014
	Coelodonta antiquitatis	Woolly Rhinoceros	Extinct; no modern analogue	Boeskorov 2001, 2004, 2011
	Ovibos moschatus	Pleistocene Musk-ox	Extinct in the region; re-introduced in tundra	Kahlke 2014
	Bison priscus	Pleistocene Bison	Extinct; modern analogue: *Bison bison*	Boeskorov 2004
	Equus lenensis	Lena Horse	Extinct; modern analogue (*Equus ferus caballus*) as livestock	Boeskorov 2004
	Equuus hemionus	Onager	Extinct in the region	Boeskorov 2004
	Alces alces	Moose	Range reduced to taiga	Meiri et al. 2020; Pavlinov et al. 2002
	Rangifer tarandus	Caribou	Range reduced to tundra	Boeskorov 2004
	Bos mutus	Yak	Extinct in the region	Dubrovo 1957
	Cervus canadensis sibiricus	Altai wapiti, Elk	Extinct in the region	Zimov et al. 2012
	Saiga tatarica borealis	Pleistocene Saiga	Extinct; modern analogue in zonal steppes of Eurasia	Boeskorov 2004; Baryshnikov and Tikhonov 1994
Small herbivores				
	Lepus timidus	Alpine hare	Range reduced to tundra	Boeskorov 2004
	Dicrostonyx spp., Lemmus spp.	Lemmings	Range reduced to tundra	Boeskorov 2004
	Urocitellus parryi	Arctic Ground Squirrel	Extant	Faerman et al. 2017; Pavlinov et al. 2002
Carnivores				
	Panthera spelaea	Cave Lion	Extinct; no modern analogue	Boeskorov 2004; Stuart and Lister 2011
	Ursos arctos	Cave Bear	Extant (as Brown Bear)	Boeskorov 2004; Sher et al. 2011
	Canis lupus	Grey Wolf	Extant	Boeskorov 2004; Pavlinov et al. 2002
	Gulo gulo	Wolferine	Extant	Boeskorov 2004; Pavlinov et al. 2002

Alopex lagopus	Arctic Fox	Range reduced to tundra	Boeskorov 2004
Vulpes vulpes	Red Fox	Extant	Boeskorov 2004; Pavlinov et al. 2002

1.1. Study area

Climate in Yakutia is highly continental, characterized by very low winter temperatures and relatively high summer temperatures (Figure S1_1). Temperatures are less extreme in Chersky, which is closer to the Arctic Sea. Here, summer precipitation of July and August is well above the temperature curve in the standard Walter-Lieth climate diagram, while in Yakutsk and Verkhoyansk conditions are closer to summer droughts.

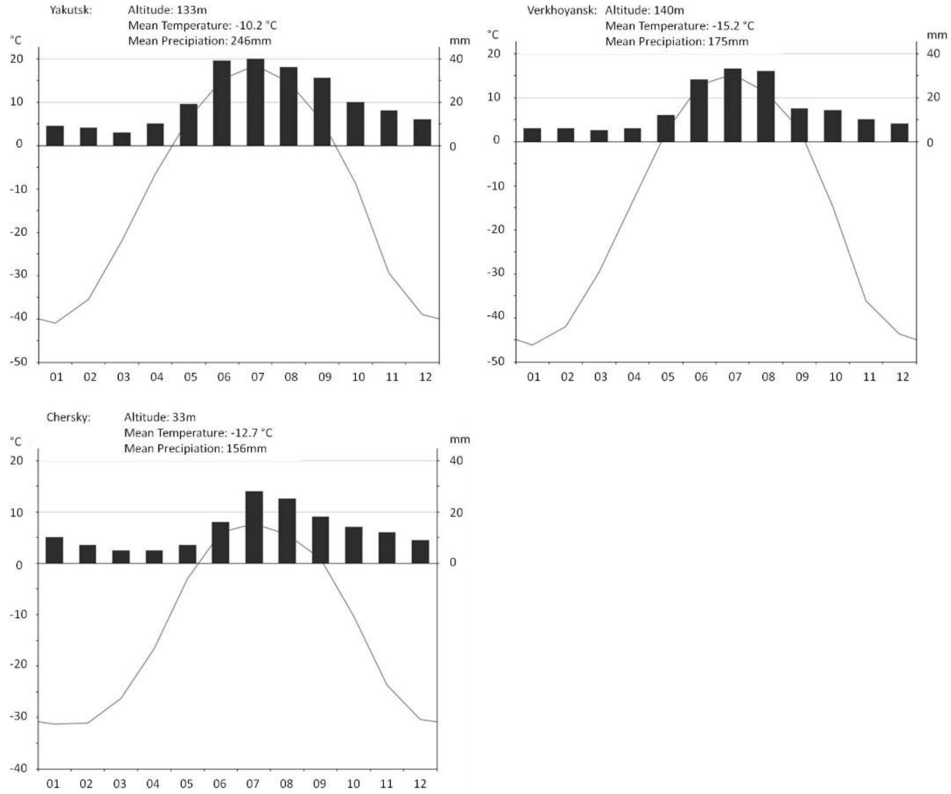

Figure S1_1. Climate diagrams of Yakutsk, Verkhoyansk and Chersky, Sakha, Russia. Modified from climate-data.org.

In Yakutia, typical steppe vegetation (*Cleistogenetea squarrosae*) is only found at sites with special microclimatic conditions (Reinecke et al., 2017): on more or less steep, SW-exposed slopes. Tundra steppes (*Carici rupestris - Kobresietea bellardii*) were restricted to soil-disturbed hilltops north of the tree line (near Pokhodsk). The number of available sites to study extrazonal steppe vegetation was especially restricted in Chersky, and all of these were out of reach of the large grazers of the Pleistocene Park nearby.

Productivity of vegetation as approximated by above-ground standing biomass (dry weight) in our study regions (see Figure S1_2) ranges between 5 and 62 g/ 40x40 cm²;

thus 3.1 to 38.8 g/ m² or 30-400 kg/ ha, with lowest values in the most continental Yana region. Productivity also depends much on habitat type, with meadows and wetlands being most productive and a wide range of productivity values being found in steppes, depending on whether they belong to densely vegetated meadow steppes or sparsely vegetated typical steppes on steep slopes.

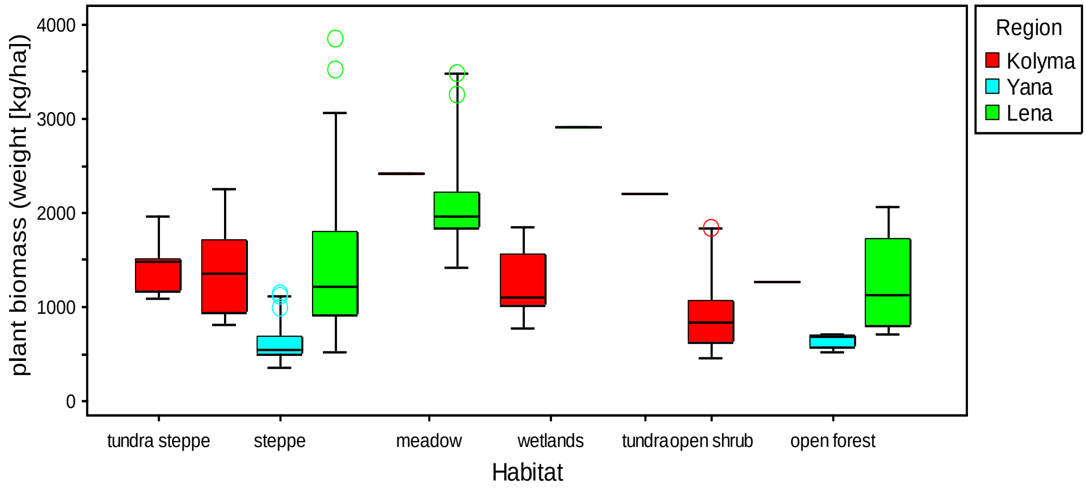

Figure S1_2. Productivity of vegetation across the study area; given as mean weight of harvested plant biomass extrapolated from subplot of 40x40 cm² per region and vegetation type.

1.2. Field sampling

Cattle and horses roamed freely around villages and were not fenced in or herded, thus mimicking natural grazing conditions. Droppings, trails and resting places indicated regular use of study sites by grazers, but at least during our study period floodplain meadows seemed to be the preferred pastures in all regions. Scrub and forest were mostly used as resting places or were frequented during roaming between pastures and had droppings along the way. Small clearings (from cutting, fire or tree fall) with higher herb cover were also occasionally grazed.

Quantification of grazing intensities in this situation was difficult, which is a common problem in rangeland ecology whenever it comes to open range or even mobile grazing systems. Herbivore density of free roaming livestock would be somewhere between that of the Bisonary (where it is highest with 17,7 t/ km²) and the large enclosure of the Pleistocene Park (where it is lowest with 1,0 t/ km²) in the larger vicinity of villages. Thus, we decided to base our analysis on dung cover. Dung was found to be spread out

evenly across the grazed areas and seemed to best reflect the intensity of grazing of different sites (Figure S1_3).

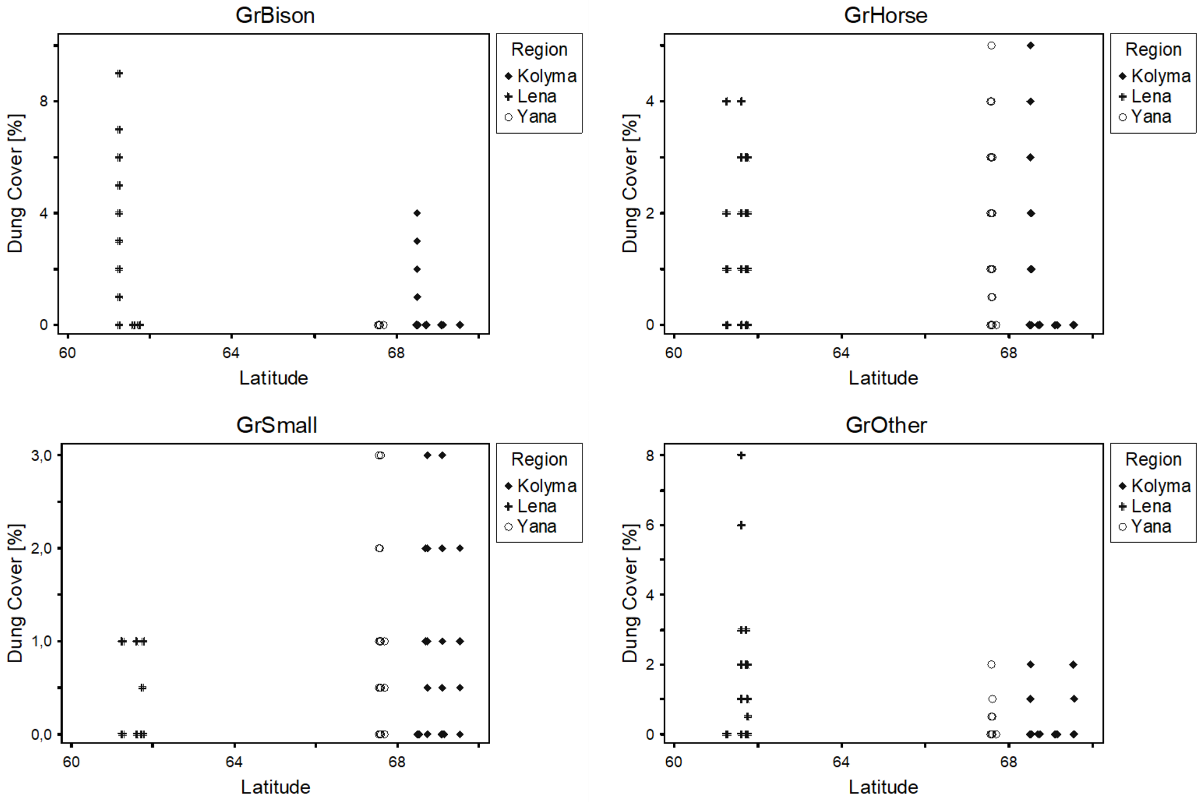

Figure S1_3: Scatterplots of dung cover in % per grazing herbivore **(bison, horse, small mammals, other large herbivores like cattle, musk ox and moose)** in all plots, grouped by study region.

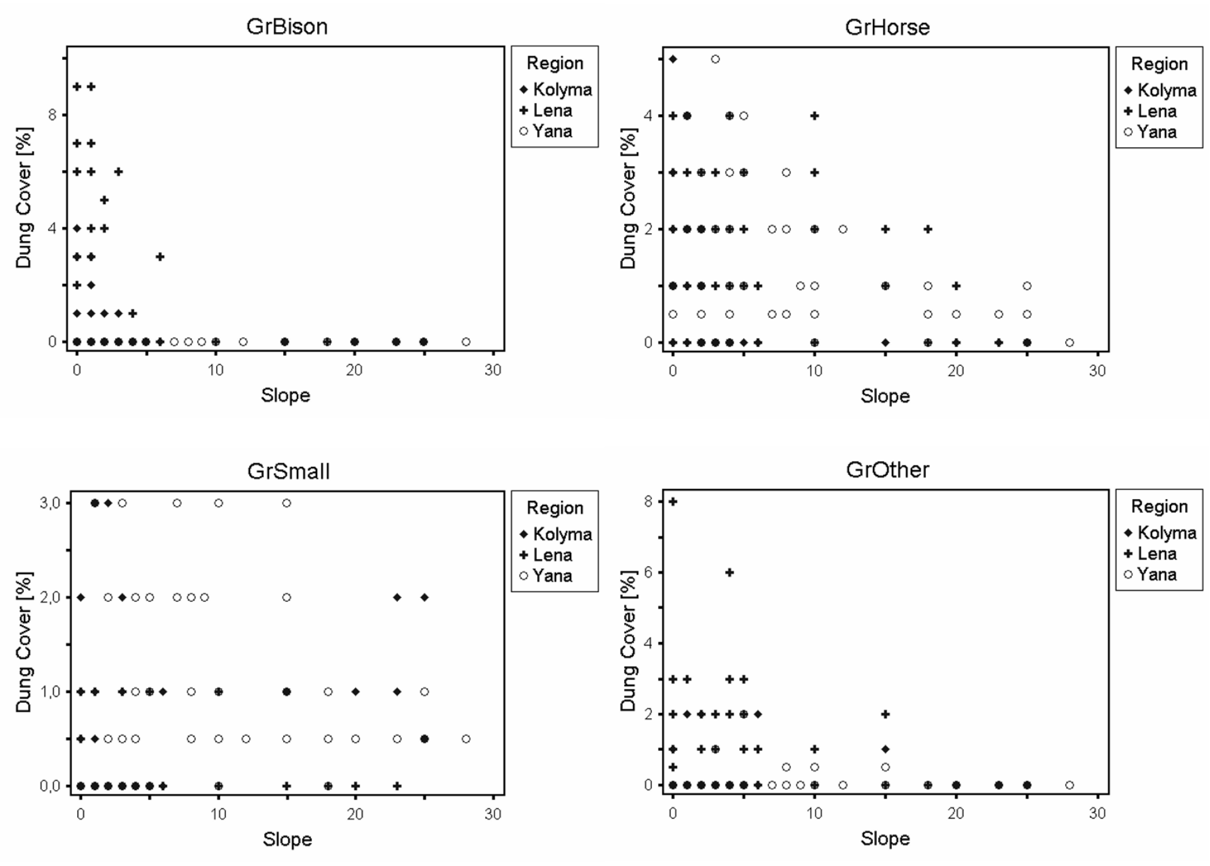

Figure S1_4: Scatterplots of dung cover in % per grazing herbivore (bison, horse, small mammals, other large herbivores like cattle, musk ox and moose) in all plots versus slope inclination, grouped by study region.

We measured 22 grazing-related plant functional traits; (Table 2; following Cornelissen et al., 2003) for a subset of 92 steppe plots (Traitset 1 and 2; Table S1_3), and 14 traits for the overall set of plots (Traitset 1; Table 3), including other vegetation types. Species for which traits had been measured usually cover more than 80 % of the biomass of each plots (herb and dwarf shrub layer), except for 16 plots for which not 80 % but still the traits for the majority of species biomass were obtained. We usually used the mean trait values of 5-10 individually measured plants. In order to complete and cross-check our list of traits for 217 species (92 steppe species, respectively) we added single measurements from BIOLFLOR (Kühn et al., 2004), TRY (Kattge and et al., 2011), GrassBase (Clayton, W.D., Vorontsova, M.S., Harman, K.T. and Williamson, 2006), Flora of Siberia (Malyschev, 2006), Flora of China (Brach and Song, 2006), Flora of the Canadian Arctic Archipelago (Aiken, S.G., Dallwitz, M.J., Consaul, L.L., McJannet, C.L., Boles, R.L., Argus, G.W., Gillett, J.M., Scott, P.J., Elven, R., LeBlanc, M.C., Gillespie, L.J., Brysting, A.K., Solstad, H., and Harris, 2007) and Forage Plants of Mongolia (Jigjidsuren and Johnson, 2003). In a few cases (<10 values), trait data was missing and we thus had to use nearest neighbor imputation to fill out single missing values.

Table S1_2. Functional traits and their attributes related to grazing with respective function and hypothesized mechanisms.

Trait	Attributes related to grazing	Function	Hypothesized mechanism	Reference
Above cover density (ACD)	High	Avoidance	Shoot attenuation as result of changing plant structure	McIntyre et al. 1999, Wesuls et al. 2012
Blade fragmentation	Compound	Tolerance	Lower loss of biomass per bite	Wesuls et al. 2012
Clonality	e.g. Rhizomes	Tolerance	Regrowth capacity	Cornelissen et al. 2003
Defense mechanisms	Thornes, spines, hairiness, secondary compounds, leaf toughness	Avoidance	Deterrence of grazers	Cornelissen et al. 2003, Wesuls et al. 2012
Fraction belowground	High/ Low	Avoidance/ Tolerance	Re-allocation below-/ aboveground (depending on vegetation type)	Wesche et al. 2012
Growth form	Prostate, rosettes, stoloniferous	Avoidance	Buds for regrowth located close to the ground	Landsberg et al. 1999, Cornelissen et al. 2003, Díaz et al. 2007, Wesuls et al. 2012
Inflorescence height	Low (protected in foliage)	Avoidance	Spatial evasion of grazing from above	Landsberg et al. 1999
Leaf size	Small		Lower loss of biomass per bite	Landsberg et al. 1999, Díaz et al. 2001, Vesk et al. 2004
Life form	Therophytes; chamaephytes	Avoidance	Temporal evasion of grazing and use of establishment opportunities; unpalatability	Cornelissen et al. 2003
Longevity	Annual	Avoidance	Temporal evasion of grazing; use of establishment opportunities	Díaz et al. 2001, 2007, Vesk et al. 2004
Growth type	Herbs; shrubs	Avoidance	Lower palatability than grasses	Vesk et al. 2004, Díaz et al. 2007, Wesuls et al. 2012
Resprouting capacity	Low meristems, belowground storage organs	Tolerance	Regrowth capacity	Landsberg et al. 1999, Cornelissen et al. 2003
Root type	Tap root	Avoidance	Resistance to trampling	Landsberg et al. 1999
Seed mass	Small	Avoidance	High establishment potential	Vesk et al. 2004
Seed productivity	Many	Avoidance	High establishment potential	Vesk et al. 2004
Shoot diameter	Large (for woody shoots)	Tolerance	Re-allocation to side instead of high growth	Gill 2006
Shoot height	Short	Avoidance	Spatial evasion of grazing from above	Landsberg et al. 1999, Díaz et al. 2001, 2007, Cornelissen et al. 2003
Specific Leaf Area (SLA)	High/ Low	Tolerance/ Avoidance	Fast regrowth resulting in tender leaves/ lower palatability due to leaf toughness	Díaz et al. 2001, Vesk et al. 2004, Wesuls et al. 2012
Stem/ Leaf ratio	Stemmy	Avoidance	Low palatability	Landsberg et al. 1999, Wesuls et al. 2012

Table S1_3. Overview on plant functional traits measured.

abbreviation	type	trait	value
			TRAITSET 1
**abbreviation**	**type**	**trait**	**value**
**PlantType**	**Qual.**	**plant type**	herb; grass; dwarfshrub
**Long**	**Qual.**	**longevity**	annual; perennial
**LF**	**Qual.**	**life form**	hemikryptophyte; chamaephyte; phanerophyte; therophyte;
**Grow**	**Qual.**	**growth form**	erect leafy; short basal; rosette; long basal; cushion; tussock;
**St/L**	**Qual.**	**stem/ leaf ratio**	stemmy; moderately leafy; leafy
**Leaf**	**Qual.**	**blade fragmentation**	broad & entire; broad & compound; long & flat; long & closed
**Clon**	**Qual.**	**clonality**	none; aboveground; belowground; aboveground & belowground
**Rhizome**	**Qual.**	**rhizome**	yes; no
**D**	**Qual.**	**defenses**	none; thorny/ spiky; tomentous; chemical; leathery; other
**Root**	**Qual.**	**root type**	taproot; several main roots
**ShootHeight**	**Quant.**	**shoot height**	[cm]
**LeafLength**	**Quant.**	**leaf length**	[cm]
**LeafWidth**	**Quant.**	**leaf width**	[cm]
**LeafRatio**	**Quant.**	**leaf ratio**	[cm]
			TRAITSET 2
	**type**	**trait**	**Value**
	**Quant.**	**fraction belowground**	[%]
	**Quant.**	**infloresence height**	[cm]
	**Quant.**	**relative infloresence**	[cm]
	**Quant.**	**shoot diameter**	[cm]
	**Quant.**	**ACD**	[%]
	**Quant.**	**SLA**	[mm^2/mg]
	**Quant.**	**root length**	[cm]
	**Qual.**	**seed productivity**	few; several; many; abundant

We collected biomass and soil samples from each vegetation plot. In grassland habitats (meadow, steppe, tundra steppe) three subplots with a size of 40 x 40 cm^2 were randomly selected across the vegetated area of each plot to account for spatial variability. We took one soil and one plant biomass sample per subplot in these habitats. In forest, scrub and tundra habitats, only one soil sample and no biomass samples were taken per plot. Aboveground biomass of each subplot was cut approximately 1 cm above ground using scissors, excluding dead standing biomass, and then air dried. We sampled the topsoil below the litter layer using a 100 cm^3 core cutter.

Slope inclination was estimated in the field as percent inclination. Slope aspect (N, NE, E, SE, S, SW, W, NW) was measured in the field, using a Garmin GPS (Garmin GPSMAP 64s) by walking straight downhill for a few meters until the direction was reliably given. In addition, we checked google earth maps of the location for correctness. We then derived northernness and easternness from aspect in degrees (360°; with 0°=N, 90°=E) by taking the cosine (northerness) and sine (easterness), thus transforming the degrees to a value between -1 and 1.

Heat load was calculated according to McCune (2007). This variable estimates temperatures on a land surface, based on the amount of potential direct incident radiation (DIR), which depends on latitude, slope aspect and slope inclination, while taking into account the time of the day that surface is subjected to this radiation.

The intensity of grazing by each grazing animal (bison, horse, cattle, small mammals) was estimated based on the density of droppings within each vegetation plot (in %). Small mammals were mostly represented by ground squirrels (*Urocitellus parryii*). Defecation is considered part of a grazing effect (fertilization), apart from the actual intake of plant biomass. Other proxies, which have proven useful in other studies, for example in Mongolia and Tibet, like distance to town or water well, did not work in our setting, as livestock was not bound to settlements (except cattle to some degree). We also estimated approximate intensity of grazing in the field, but the simple index (high, medium, low) we developed from this information did also not prove useful. Instead, dung density was crucially evaluated already in the field and found to be the best approximation for grazing intensity, even when considering animal movement. Other studies have also shown that dung density is a useful indicator (e.g. Wang et al. 2018).

Macroclimatic variables (Bio 1/ 7/ 10/ 12/ 15/ 18/ 19) were extracted from WorldClim (Hijmans et al., 2005). Annual Mean Temperature (Bio 1) and Annual Mean Precipitation (Bio 12) give basic information on climate; Mean Temperature of Warmest Quarter (Bio 10) and Precipitation of Warmest Quarter (Bio 18) give information on vegetation-relevant summer conditions; and Temperature Annual Range (Bio 7) and Precipitation Seasonality (Bio 15) give information on seasonal differences in climate, thus its continentality. We used GPS coordinates of plots to extract the spatially explicit climate data from the WorldClim model.

1.3. Sample processing

Soil samples were initially dried in the lab for 48 hours at 40°C. Samples were then sieved using a 2 mm coarse screen, using the fine material for further analysis. We measured pH (H_2O) and electric conductivity (EC) after 1h and 24 h. We measured the C/N ratio through combustion in a CN analyzer (Vario Elementar). To assess the amount of plant available nutrients (Ca, Mg, K, P) we prepared soil extractions following the Olsen P method (Sims, 2000). Nutrient contents in these extractions were measured by spectrometry (ICP-OES,

Institute of Soil Science, Hannover University). Rest water was measured after drying of samples at 105°C for 24h. The carbonate content was first assessed with a quick test using 10% HCl, and samples showing a reaction were further analyzed using a calcimeter following Scheibler's method (ON L 1084-99, 1999). Rest water content was used to calibrate nutrient contents per g soil and carbonate content to correct C/N measurements.

Plant biomass was cut into pieces of 1-3 cm length using ceramic scissors and then separated about 2 (1-3; depending on amount of plant material) times using a dividing cross. A mixed sample of the biomass was then finely ground (Leuphana University of Lüneburg; Umweltanalytisches Labor, IHI Zittau, University of Dresden). C/N-ratio was analyzed using the same procedure like the soil samples. For nutrient content we decomposed the ground plant material, using microwave decomposition (samples of 2014; Umweltanalytisches Labor, IHI Zittau, University of Dresden) and pressure decomposition (samples of 2015; Lab of the Botany Department, Senckenberg Görlitz). The decomposed biomass solution was then also analyzed by spectrometry (ICP-OES, again Hannover). The measurements of the microwave decomposition were finally converted to be comparable to measurements of pressure decomposition using linear regressions.

1.4. References

Aiken, S.G., Dallwitz, M.J., Consaul, L.L., McJannet, C.L., Boles, R.L., Argus, G.W., Gillett, J.M., Scott, P.J., Elven, R., LeBlanc, M.C., Gillespie, L.J., Brysting, A.K., Solstad, H., and Harris, J.G., 2007. Flora of the Canadian Arctic Archipelago: Descriptions, Illustrations, Identification, and Information Retrieval [WWW Document]. NRC Res. Press. Natl. Res. Counc. Canada, Ottawa. URL http://nature.ca/aaflora/data (accessed 8.30.17).

Baryshnikov, G., Tikhonov, A., 1994. Notes on Skulls of Pleistocene Saiga of Northern Eurasia. Hist. Biol. 8, 209–234. https://doi.org/10.1080/10292389409380478

Boeskorov, G.G., 2004. The North of Eastern Siberia: Refuge of Mammoth fauna in the

Holocene. Gondwana Res. 7, 451–455. https://doi.org/10.1016/S1342-937X(05)70796-6

Boeskorov, G.G., Lazarev, P.A., Sher, A. V., Davydov, S.P., Bakulina, N.T., Shchelchkova, M. V., Binladen, J., Willerslev, E., Buigues, B., Tikhonov, A.N., 2011. Woolly rhino discovery in the lower Kolyma River. Quat. Sci. Rev. 30, 2262–2272. https://doi.org/10.1016/j.quascirev.2011.02.010

Brach, A.R., Song, H., 2006. eFloras: New directions for online floras exemplified by the Flora of China Project. Taxon 55, 188–192. https://doi.org/10.2307/25065540

Clayton, W.D., Vorontsova, M.S., Harman, K.T. and Williamson, H., 2006. GrassBase - The Online World Grass Flora [WWW Document]. URL http://www.kew.org/data/grasses-db.html (accessed 8.30.17).

Cornelissen, J.H.C., Lavorel, S., Garnier, E., Diaz, S., Buchmann, N., Gurvich, D.E., Reich, P.B., Ter Steege, H., Morgan, H.D., Van Der Heijden, M.G.A., Pausas, J.G., Pooter, H., 2003. A handbook of protocols for standardised and easy measurement of plant functional traits worldwide. Aust. J. Bot. 51, 335–380. https://doi.org/10.1071/BT02124

Díaz, S., Lavorel, S., McIntyre, S., Falczuk, V., Casanoves, F., Milchunas, D.G., Skarpe, C., Rusch, G., Sternberg, M., Noy-Meir, I., Landsberg, J., Zhang, W., Clark, H., Campbell, B.D., 2007. Plant trait responses to grazing - A global synthesis. Glob. Chang. Biol. 13, 313–341. https://doi.org/10.1111/j.1365-2486.2006.01288.x

Díaz, S., Noy-meir, I., Cabido, M., 2001. Can grazing of herbaceous plants be predicted response from simple vegetative traits? J. Appl. Ecol. 38, 497–508.

Dubrovo, I., 1957. Der erste Nachweis des fossilen Yak im nördlichen Ostsibirien (Jakutien).

Vertebr. Palasiat. 1, 293–300.

Faerman, M., Bar-Gal, G.K., Boaretto, E., Boeskorov, G.G., Dokuchaev, N.E., Ermakov, O.A., Golenishchev, F.N., Gubin, S. V., Mintz, E., Simonov, E., Surin, V.L., Titov, S. V., Zanina, O.G., Formozov, N.A., 2017. DNA analysis of a 30,000-year-old Urocitellus glacialis from northeastern Siberia reveals phylogenetic relationships between ancient and present-day arctic ground squirrels. Sci. Rep. 7. https://doi.org/10.1038/srep42639

Gill, R., 2006. The influence of large herbivores on tree recruitment and forest dynamics, in: Danell, K., Bergström, R., Duncan, P., Pastor, J. (Eds.), Large Herbivore Ecology, Ecosystem Dynamics and Conservation. Cambridge University Press, Cambridge, pp. 170–202. https://doi.org/10.1017/CBO9780511617461.008

Hijmans, R.J., Cameron, S.E., Parra, J.L., Jones, P.G., Jarvis, A., 2005. Very high resolution interpolated climate surfaces for global land areas. Int. J. Climatol. 25, 1965–1978. https://doi.org/10.1002/joc.1276

Jigjidsuren, S., Johnson, D., 2003. Forage plants in Mongolia. Admon Publishing, Ulaanbaatar.

Kahlke, R.D., 2014. The origin of Eurasian Mammoth Faunas (Mammuthus-Coelodonta Faunal Complex). Quat. Sci. Rev. 96, 32–49. https://doi.org/10.1016/j.quascirev.2013.01.012

Kattge, J., et al., 2011. TRY - a global database of plant traits. Glob. Chang. Biol. 17, 2905–2935. https://doi.org/10.1111/j.1365-2486.2011.02451.x

Kühn, I., Durka, W., Klotz, S., 2004. BiolFlor - A new plant-trait database as a tool for plant

invasion ecology. Divers. Distrib. 10, 363–365. https://doi.org/10.1111/j.1366-9516.2004.00106.x

Landsberg, J., Lavorel, S., Stol, J., 1999. Grazing response groups among understorey plants in arid rangelands. J. Veg. Sci. 10, 683–696.

Malyschev, L.I., 2006. Flora of Siberia. Science Publishers, U.S.

McCune, B., 2007. Improved estimates of incident radiation and heat load using non-parametric regression against topographic variables. J. Veg. Sci. 18, 751–754. https://doi.org/10.1658/1100-9233(2007)18%5B751:IEOIRA%5D2.0.CO;2

McIntyre, S., Lavorel, S., Landsberg, J., Forbes, T.D.A., 1999. Disturbance response in vegetation - towards a global perspective on functional traits. J. Veg. Sci. 10, 621–630. https://doi.org/10.2307/3237077

Meiri, M., Lister, A., Kosintsev, P., Zazula, G., Barnes, I., 2020. Population dynamics and range shifts of moose (Alces alces) during the Late Quaternary. J. Biogeogr. 47, 2223–2234. https://doi.org/10.1111/jbi.13935

Pavlinov, I.Ya., Kruskop, S.V., Varshavsky, A.A., Borisenko, A.V., 2002. Nazemnye zveri Rossii (Terrestrial mammals of Russia [In Russian]). Publishing Hous KMK, Moscow.

Reinecke, J., Troeva, E., Wesche, K., 2017. Extrazonal steppes and other temperate grasslands of northern Siberia - Phytosociological classification and ecological characterization. Phytocoenologia 47, 167–196. https://doi.org/10.1127/phyto/2017/0175

Sher, A. V., Weinstock, J., Baryshnikov, G.F., Davydov, S.P., Boeskorov, G.G., Zazhigin, V.S., Nikolskiy, P.A., 2011. The first record of “spelaeoid” bears in Arctic Siberia. Quat.

Sci. Rev. 30, 2238–2249. https://doi.org/10.1016/j.quascirev.2010.10.016

Sims, J.T., 2000. Soil test phosphorus: Olsen P. Methods phosphorus Anal. soils, sediments, residuals, waters 20.

Stuart, A.J., Lister, A.M., 2011. Extinction chronology of the cave lion Panthera spelaea. Quat. Sci. Rev. 30, 2329–2340. https://doi.org/10.1016/j.quascirev.2010.04.023

Vesk, P.A., Leishman, M.R., Westoby, M., 2004. Simple traits do not predict grazing response in Australian dry shrublands and woodlands. J. Appl. Ecol. 41, 22–31. https://doi.org/10.1111/j.1365-2664.2004.00857.x

Wesche, K., Krause, B., Culmsee, H., Leuschner, C., 2012. Fifty years of change in Central European grassland vegetation: Large losses in species richness and animal-pollinated plants. Biol. Conserv. 150, 76–85. https://doi.org/10.1016/j.biocon.2012.02.015

Wesuls, D., Oldeland, J., Dray, S., 2012. Disentangling plant trait responses to livestock grazing from spatio-temporal variation: The partial RLQ approach. J. Veg. Sci. 23, 98–113. https://doi.org/10.1111/j.1654-1103.2011.01342.x

Zimov, S.A., Zimov, N.S., Tikhonov, A.N., Chapin, I.S., 2012. Mammoth steppe: A high-productivity phenomenon. Quat. Sci. Rev. 57, 26–45. https://doi.org/10.1016/j.quascirev.2012.10.005

2.

Appendix S2. Supplementary information on results

Table S2_1. List of abbreviations for plant traits and biomass/ soil data.

Abbreviation	Trait	Abbreviation	Variable
LF thero	Life form: therophyte	S_	Soil variable
LF hemicrypto	Life form: hemicryptophyte	B_	Biomass variable
LF chamae	Life form: chamaephyte	Weight	Weight in [g]
D none	Defenses: none	Fine	Weight of fine soil [g]
D hairy	Defenses: hairy	Coarse	Weight of course soil [g]
D chem	Defenses: chemical	RestWater	Rest water content in [%/ g soil]
Grow cushion	Growth form: cushion	pH	pH after 24 hours
Grow shBasal	Growth form: short basal	EC	Electric conductivity after 24 hours
Grow semiBasal	Growth form: semi basal	CaCO3	Carbonate content in [g/ kg soil]
Grow rosette	Growth form: rosette	C%	Percent carbon content
Root tap	Root: Tap root	N%	Percent nitrogen content
ST/L stemmy	Stem-Leaf ratio: stemmy	C/N	Ratio of C and N
Leaf closed	Blade fragmentation: long & closed	Ca	[g/kg]
FlowerHeight	Flowering height	Mg	[g/kg]
CovLitter	Cover litter layer	K	[g/kg]
CovHerb	Cover herb layer	P	[g/kg]
CovS2	Cover dwarf shrub layer		
CovGround	Cover open ground		
CovRock	Cover rocks		

Table S2_2. Results of variation partitioning of biomass data.

		**Overall**		**Steppes only**	
**Ordination method**	**Environmental variable**	**variance**	**% explained**	**variance**	**% explained**
**Variation Partitioning (pCCAs, Unique effects)**					
	- Soil	0.19	18.7	0.10	10.0

	- Macroclimate	0.18	17.7	0.10	9.7
**Variation Partitioning (pCCAs, Unique effects)**					
	- Soil	0.12	12.1	0.10	10.3
	- Macroclimate	0.16	16.3	0.10	10.4
	- Microclimate	0.02	1.8	0.01	1.4
**Variation Partitioning (pCCAs, Unique effects)**					
	- Soil	0.15	15.5	0.11	10.7
	- Macroclimate	0.18	18.2	0.09	8.7
	- Grazing	0.02	1.6	0.01	1.3

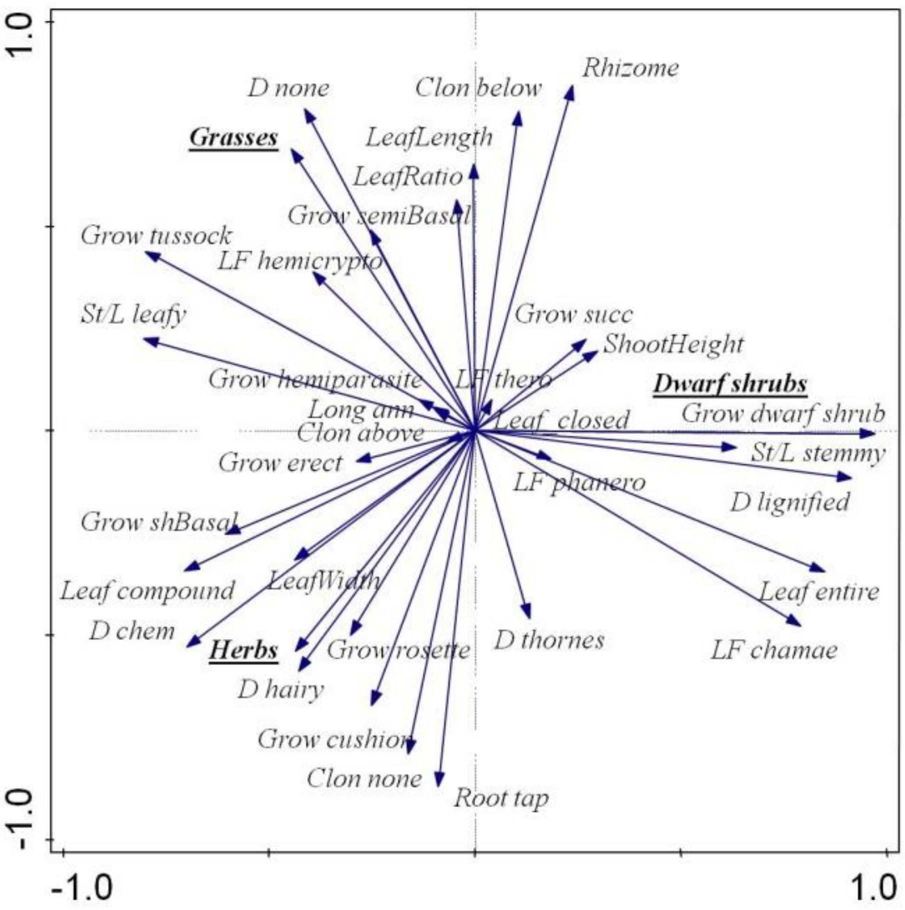

Figure S2_1. PCA of community weighted means of plant traits, which cluster around life forms dwarf shrubs, grasses and herbs (Eigenvalues: Axis1: 0.25, Axis2: 0.19).

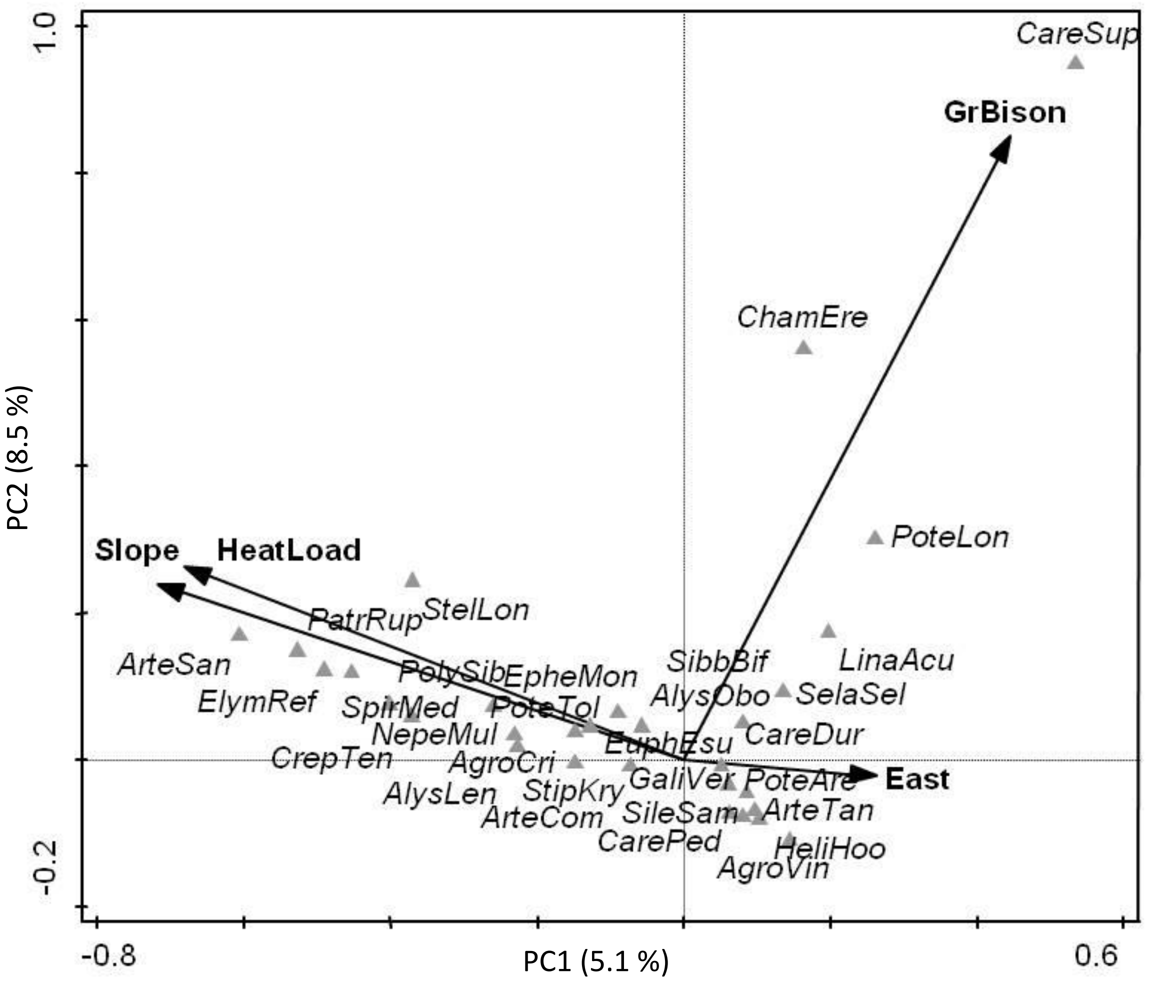

Figure S2_2. pCCa with macroclimate as co-variable and significant microclimate (slope inclination - Slope; heat load - HeatLoad; easternness - East) and grazing variables (bison - GrBison; forward selection, 499 permutations) for only steppe plots; total inertia: 3.8, Eigenvalues: Axis1: 0.20, Axis2: 0.13; %explained variance: Axis1: 5.1, Axis2: 3.3; only most abundant species used and 40 best fitted species shown; species' cover square root-transformed.

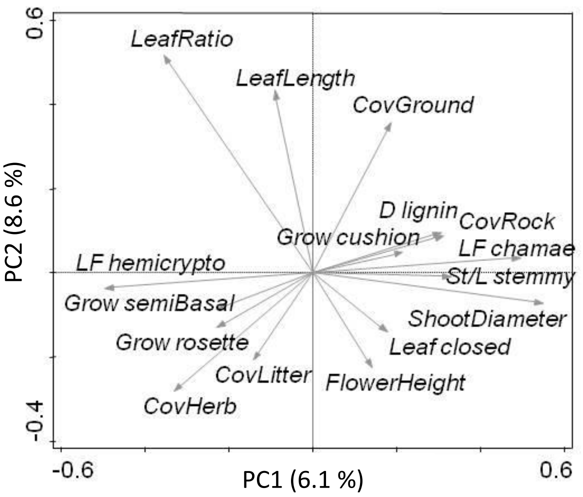

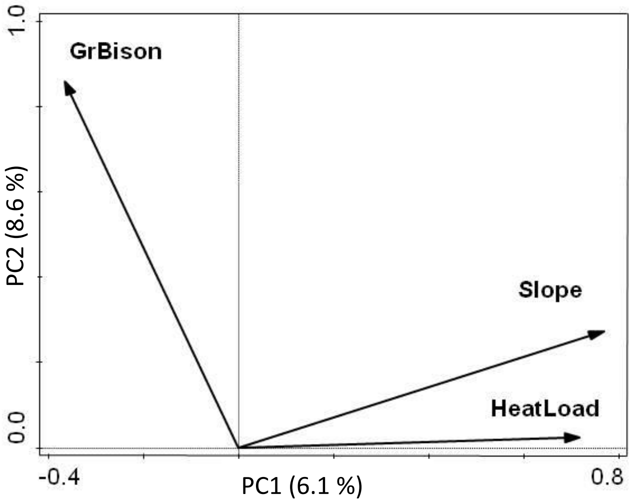

Figure S2_3. CWM-RDA (forward selection, 499 permutations) of steppe data set; total variance: 3843, explained variance: 10.0 %; Eigenvalues: Axis1: 0.05, Axis2: 0.02; %explained variance: Axis1: 6.1, Axis2: 2.5; a) CWMs (only 50 % most significant traits, which are also confirmed by direct univariate correlation of CWMs with environmental variables, are shown), b) significiant microclimate (slope inclination (Slope); heat load (HeatLoad)) and grazing variables (bison (GrBison)).

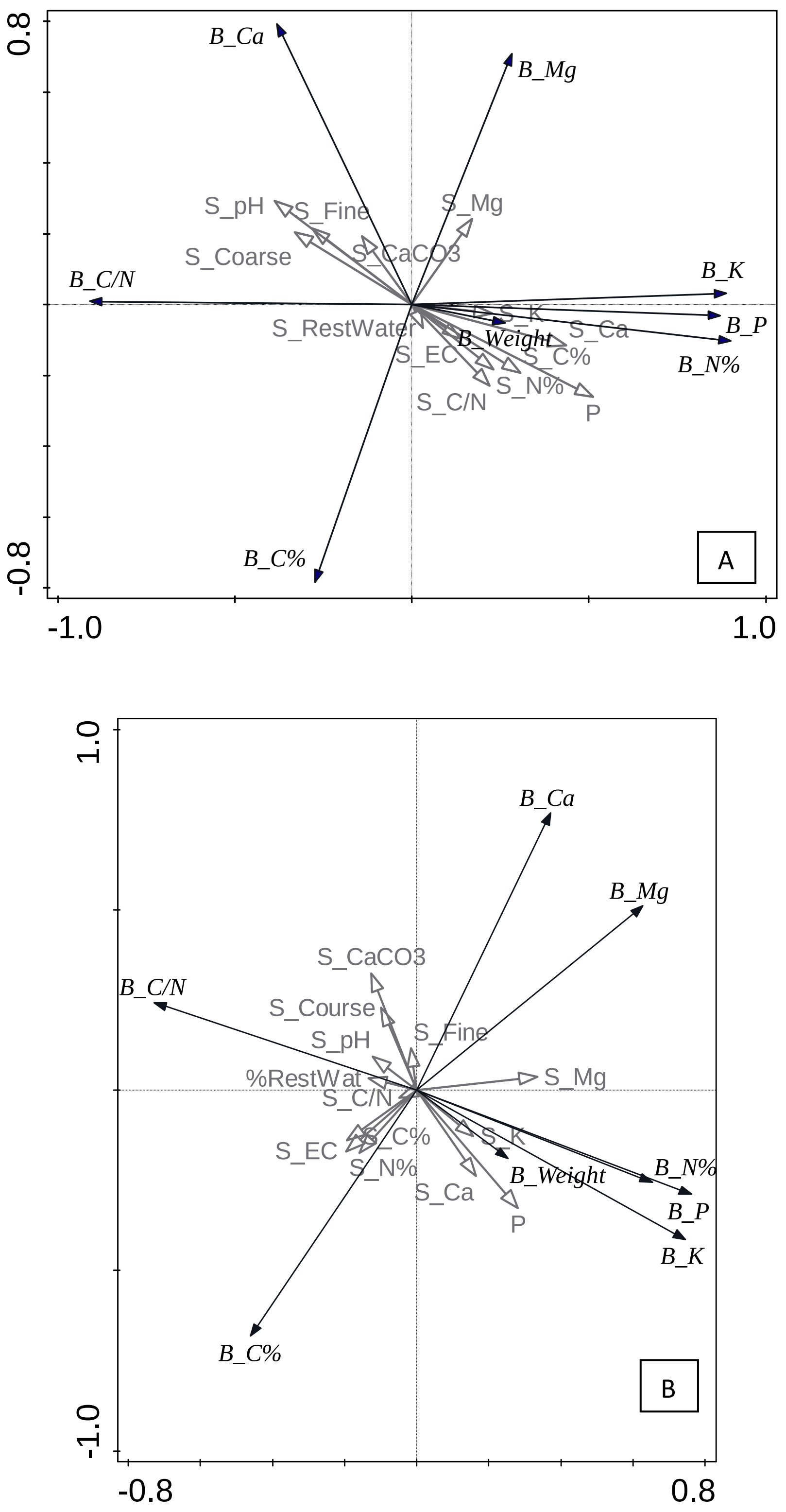

Figure S4_4. Correlation-matrix PCA of biomass variables (centered and standardized) with post hoc fitted soil variables for a) overall data; total variance: 1200, explained variance: 40.7 %, Eigenvalues: Axis1: 0.44, Axis2: 0.22; and b) for steppes; total variance: 720, explained variance: 37.5 %, Eigenvalues: Axis1: 0.36, Axis2: 0.22. See Table 1 for abbreviations.

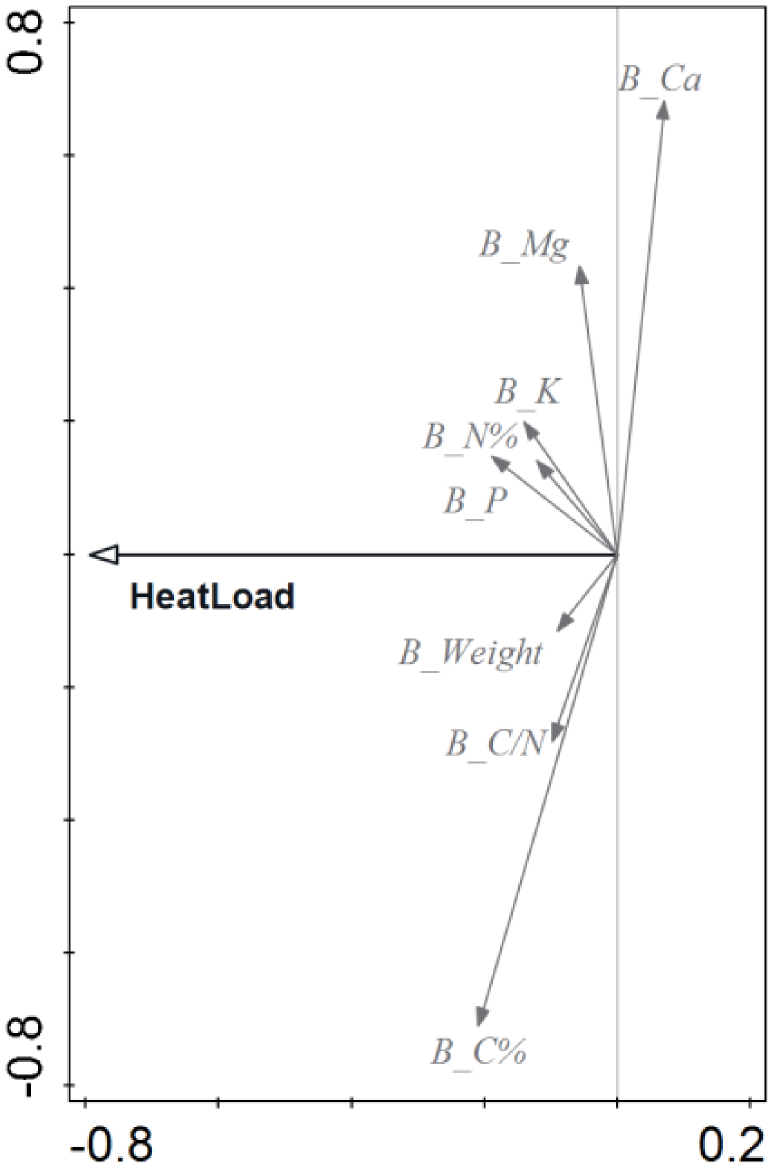

Figure S2_5. Biomass-RDA (forward selection, 499 permutations) of steppe data set, total variance: 417, explained variance: 3.0 %, Eigenvalues: Axis1: 0.02, Axis2: 0.02; %explained variance: Axis1: 3.0, Axis2: 28.8; only one variable was significant (HeatLoad = heat load) and is shown. See Table 1 for abbreviations.
